# DNA Methylation Profiles and Their Relationship with Cytogenetic Status in Adult Acute Myeloid Leukemia

**DOI:** 10.1371/journal.pone.0012197

**Published:** 2010-08-16

**Authors:** Sara Alvarez, Javier Suela, Ana Valencia, Agustín Fernández, Mark Wunderlich, Xabier Agirre, Felipe Prósper, José Ignacio Martín-Subero, Alba Maiques, Francesco Acquadro, Sandra Rodriguez Perales, María José Calasanz, Jose Roman-Gómez, Reiner Siebert, James C. Mulloy, José Cervera, Miguel Angel Sanz, Manel Esteller, Juan C. Cigudosa

**Affiliations:** 1 Molecular Cytogenetics Group, Centro Nacional Investigaciones Oncologicas (CNIO), Centro de Investigaciones de Enfermedades Raras (CIBERER), Madrid, Spain; 2 Hematology Service, Hospital Universitario La Fe, Valencia, Spain; 3 Cancer Epigenetics and Biology Program, Bellvitge Institute for Biomedical Research-Catalan Institute of Oncology (IDIBELL-ICO), Barcelona, Spain; 4 Division of Experimental Hematology, Cincinnati Children's Hospital Medical Center, University of Cincinnati College of Medicine, Cincinnati, Ohio, United States of America; 5 Division of Cancer and Area of Cell Therapy and Hematology Service, Foundation for Applied Medical Research, Clínica Universitaria, Universidad de Navarra, Pamplona, Spain; 6 Department of Genetics, University of Navarra, Pamplona, Spain; 7 Hematology Service, Hospital Universitario Reina Sofia, Córdoba, Spain; 8 Institute of Human Genetics, University Hospital Schleswig-Holstein, Campus Kiel, Christian-Albrechts University, Kiel, Germany; Deutsches Krebsforschungszentrum, Germany

## Abstract

**Background:**

Aberrant promoter DNA methylation has been shown to play a role in acute myeloid leukemia (AML) pathophysiology. However, further studies to discuss the prognostic value and the relationship of the epigenetic signatures with defined genomic rearrangements in acute myeloid leukemia are required.

**Methodology/Principal Findings:**

We carried out high-throughput methylation profiling on 116 de novo AML cases and we validated the significant biomarkers in an independent cohort of 244 AML cases. Methylation signatures were associated with the presence of a specific cytogenetic status. In normal karyotype cases, aberrant methylation of the promoter of *DBC1* was validated as a predictor of the disease-free and overall survival. Furthermore, *DBC1* expression was significantly silenced in the aberrantly methylated samples. Patients with chromosome rearrangements showed distinct methylation signatures. To establish the role of fusion proteins in the epigenetic profiles, 20 additional samples of human hematopoietic stem/progenitor cells (HSPC) transduced with common fusion genes were studied and compared with patient samples carrying the same rearrangements. The presence of *MLL* rearrangements in HSPC induced the methylation profile observed in the *MLL*-positive primary samples. In contrast, fusion genes such as *AML1/ETO* or *CBFB/MYH11* failed to reproduce the epigenetic signature observed in the patients.

**Conclusions/Significance:**

Our study provides a comprehensive epigenetic profiling of AML, identifies new clinical markers for cases with a normal karyotype, and reveals relevant biological information related to the role of fusion proteins on the methylation signature.

## Introduction

Acute myeloid leukemia (AML) is the most common type of acute leukemia in adults. Chemotherapy induces complete remission in 70 to 80 percent of patients, but half relapse and die. Therefore, accurate predictors of clinical outcome can contribute to the design of appropriate treatment for individual patients. Cytogenetic and molecular markers are currently the most powerful prognostic factors. The karyotype is used to classify patients as being at low, intermediate, or high risk. Nevertheless, there is substantial heterogeneity within each risk group. Thirty-five to 50 percent of patients have a normal karyotype, and molecular markers, such as mutations in *FLT3*, *CEBPα*, and *NMP1*, further stratify this large group. These prognostic markers are non-random irreversible genetic aberrations that activate oncogenes, inactivate tumor suppressor genes, and form novel chimeric genes that lead cells to progress to the malignant phenotype [1], [2].

There is increasing evidence that, in addition to genetic aberrations, therapeutically reversible epigenetic events play a critical role in the pathogenesis of human cancer [3], [4]. Methylation of the cytosines at the palindromic CpG sites clustered in gene promoter regions plays an important role in the epigenetic silencing of genes such as *ESR1*, *IGSF4*, and *CDKN2B*/p15 during the development, progression, and relapse of leukemia [5], [6], [7]. Furthermore, recent data suggest that promoter DNA methylation patterns could provide important additional information regarding risk and outcome [8], [9], [10]. However, the prognostic value of individual DNA methylation biomarkers, on the context of specific cytogenetic subgroups has not been evaluated.

From the biological standpoint, genome AML-associated fusion proteins that result from chromosome translocations have been reported to help establish specific DNA methylation patterns in AML. For example, *PML/RARα* and *AML1/ETO* have been shown to recruit both histone deacetylases and DNA methyltransferases to induce transcriptional repression of target genes [11], and abundant epigenetic lesions have been identified along with recurrent chromosome translocations such as t(8;21), t(15;17) and Inv(16) [8], [9]. Although these data support the idea of a link between epigenetic and genetic changes, the contribution of the fusion proteins to the aberrant DNA methylation signature need to be better established.

Here we report a detailed comprehensive methylation profile to systematically explore the epigenomic variation underlying AML. The findings were correlated with clinical outcomes, and the contribution of different chromosomal rearrangements to the methylation profile was determined.

## Materials and Methods

### Samples

Two series of patients diagnosed with de novo AML were studied. An original series of 116 cases was analyzed. DNA was collected in all instances from the leftover biological material after a proper diagnosis was achieved at the cytogenetic laboratories of the Universidad de Navarra (Pamplona, Spain), the Spanish National Cancer Center (CNIO, Madrid, Spain), and the Christian-Albrechts University (Kiel, Germany) (Table 1). The Spanish patients were treated according to the PETHEMA LAM99 clinical protocol [12]. The control samples comprised 4 bone marrow specimens and 2 CD34+ selections from the mobilized peripheral blood stem cells of healthy donors. An independent validation series of 244 cases was collected from Hospital la Fe (Valencia, Spain) and Hospital Reina Sofía (Córdoba, Spain). Thirteen cases of primary acute lymphoblastic leukemia (ALL) were also included. All samples were analyzed anonymously.

**Table 1:** Summary of clinical data and distribution according to the DNA methylation profile.

		Cluster		
		Group I	Group II	
**Gender**				
Male	63	31	32	
Female	53	27	26	
**Age**				
<60 yr	31	15	16	
>60yr	85	43	42	
**FAB Sub-type**				p* = 0.011
M0	4	3(75%)	1(25%)	
M1	19	13(68%)	6(32%)	
M2	30	16(53%)	14(47%)	
M3	9	0(0%)	9(100%)	
M4	13	8(61%)	5(39%)	
M4EO	11	5(45%)	6(55%)	
M5	27	10(37%)	17(63%)	
M6	3	3(100%)	0(0%)	
TOTAL	116	58(50%)	58(50%)	
**Cytogenetic Prognosis Group** **				p*<0.001
Favorable	30	6(20%)	24(80%)	
t(8;21)	10	1(10%)	9(90%)	
inv(16)	11	5(45%)	6(55%)	
t(15;17)	9	0(0%)	9(100%)	
Intermediate	61	32(52%)	29(48%)	
Normal Karyotype	41	23(56%)	18(44%)	
Single Trisomy	12	5(42%)	7(68%)	
Double Trisomy	3	2(67%)	1(33%)	
Other Intermediate	5	2(40%)	3(60%)	
Adverse	25	20(80%)	5(20%)	
Complex Karyotype	14	14(100%)	0(0%)	
MLL	7	2(29%)	5(71%)	
Other Adverse	4	4(100%)	0(0%)	
**FLT3**				p* = 0.537
ITD	15	9(60%)	6(40%)	
Mutation	6	2(33%)	4(67%)	
ITD+Mutation	1	1(100%)	0(0%)	
Negative	61	35(57%)	26(43%)	
ND	33	11(33%)	22(67%)	

*p value from Fisher-Freeman-Halton exact test.

**Samples were analyzed cytogenetically according to standard methods and were sub-classified into three sub-groups according to the CALGB cytogenetics cumulative incidence of relapse classification system [39]. The Fisher-Freeman-Halton exact test was calculated for the three major groups (favorable, intermediate, and adverse).

We analyzed DNA from 25 primary human hematopoietic stem cells/progenitor cells (HSPC) taken from cord blood samples. Human umbilical CB was obtained by the Translational Trials Support Laboratory at CCHMC under a protocol approved by the CCHMC Institutional Review Board. No identifying information related to the infant or mother was obtained with these collections. These HSPC were stably transduced with retroviruses expressing different fusion proteins or with an empty vector and cultured for 12 to 17 weeks. This model have been studied in depth elsewhere [13].

### Methylation profiling

All samples were processed at CNIO. Microarray-based DNA methylation profiles were obtained using the GoldenGate Methylation Cancer Panel I (Illumina, Inc., San Diego, CA, USA). The panel contains 1505 CpG sites selected from 807 genes, including oncogenes and tumor suppressor genes, imprinted genes, genes involved in various signaling pathways, and genes responsible for DNA repair, cell cycle control, metastasis, differentiation, and apoptosis.

The methylation assay was performed as described previously [14]. Briefly, for each CpG site, four probes were designed—two allele-specific oligos (ASO) and two locus-specific oligos (LSO). Each ASO-LSO pair corresponded to either the methylated or unmethylated state of the CpG site. Bisulfite conversion of DNA samples was performed using the EZ DNA methylation kit (Zymo Research, Orange, CA, USA). The remaining assay steps were identical to those of the GoldenGate genotyping assay [15], using reagents and conditions recommended by the manufacturer (Illumina, Inc). The arrays were hybridized under a temperature gradient program imaged using a BeadArray Reader (Illumina, Inc). Each methylation data point is represented by fluorescent signals from the M (methylated, Cy5) and U (unmethylated, Cy3) alleles. Background intensity, computed from a set of negative controls, was subtracted from each analytical data point. The ratio of fluorescent signals was then computed from the two alleles according to the following formula:
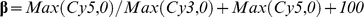
The 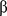 value provides a continuous measure of levels of DNA methylation in the samples, ranging from 0 in the case of completely unmethylated sites to 1 in completely methylated sites. An absolute value is used in the denominator of the formula, as a compensation for any negative values which may arise from global background subtraction (i.e.,over subtraction). A constant bias of 100 is added to the denominator to regularize 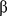 when both U and M values are small. (More setailed information could be found on the GoldenGate Assay for Methylation System User Guide; Illumina Part No.#11228975)”. The high reproducibility of the GoldenGate Methylation Cancer Panel I (mean coefficient of determination [R^2^] = 0.99) has been demonstrated elsewhere [16].

Before the methylation data were analyzed, 84 CpGs located on chromosome X and 11 CpGs showing interlab differential methylation (i.e. interarray version) were excluded to avoid possible sources of biological and technical bias. A total of 1410 CpGs from 767 genes underwent further statistical analysis [17]. Whether a CpG falls within a CpG island or not (non-clustered CpG) has been defined according to Takai and Jones relaxed criteria [18].

### Hierarchical cluster analysis and differential methylation analysis

Hierarchical clustering was performed using Gene Cluster 3.0 and visualized using Treeview 3.0 (both from http://rana.lbl.gov/eisen). Selection of differentially methylated CpGs or unmethylated CpGs was based on the presence of a Delta ß value (Δß) of at least 0.34 between samples and controls [Δß = samples mean ß value – controls mean ß value] and a false discovery rate (FDR) below 0.05, calculated using *t* tests or analysis of variance if more than two groups were compared. The data were analyzed through the use of Ingenuity Pathways Analysis (Ingenuity® Systems, www.ingenuity.com). Canonical pathways analysis identified the pathways from the Ingenuity Pathways Analysis library of canonical pathways that were most significant to the data set. The significance of the association between the data set and the canonical pathway was measured in 2 ways: 1) A ratio of the number of molecules from the data set that map to the pathway divided by the total number of molecules that map to the canonical pathway is displayed. 2) Fisher's exact test was used to calculate a p-value determining the probability that the association between the genes in the dataset and the canonical pathway is explained by chance alone.

We examined the association between each cluster identified on the unsupervised analysis and each of the karyotype, the cytogenetic prognostic group, the F.A.B subtype and the FLT3 status variables using a chi-squared test. Because of the relatively small sample size and large number of zero cells, we computed p-value using Monte Carlo simulation, with 500000 replicates, instead of relying on the asymptotic chi-squared distribution of the test statistic.

### Distribution of aberrant promoter methylation of *DBC1* and *CDKN2B* in an independent AML validation series

Methylation-specific polymerase chain reaction (MSP) analysis was used to determine the methylation status of the *DBC1* (NM_014618) and *CDKN2B* (NM_078487.2) genes in two independent experiments for all samples, as previously described [19]. DNA was extracted using the QIAmp DNA Mini Kit (Qiagen GmbH, Hilden, Germany) and modified using the EpiTect® Bisulfite Kit (Qiagen GmbH, Hilden, Germany). Primer sequences, PCR conditions, and product size are shown in Document S1. Bisulfite sequencing was performed in 9 samples as previously described [20]. Primer sequences, designed using the Methyl Primer Express Software® (Applied Biosystems Inc, Foster City, CA, USA), are shown in Document S1. At least 10 colonies of each product were sequenced. CpGs with over 40% positive clones were considered as abrerrantly methylated.

### Identification of *DBC1* as a prognostic marker

To build a predictor based on the methylation status of the 115 selected genes with larger variation across the original series, we used the web tool SignS (http://signs.bioinfo.cnio.es) [21]. To use this web tool we provided three files: one with the gene methylation data, another with the survival time, and a third indicating whether the event was observed or not (the latter being the censored cases). The method used was based on boosting with component-wise univariate Cox models. The number of boosting iterations was selected using cross-validation. For the final results, the variables (CpGs) with non-zero coefficients at the optimal number of iterations were selected. These results were validated using scores from a second final model using the threshold gradient descent method.

Survival was analyzed using Kaplan-Meier plots and the log-rank test. Cox proportional hazards models were fitted for multivariate analysis. A two-tailed p<0.05 was considered statistically significant. SSPSv.15 was used for the statistical analysis.

### Aberrant promoter methylation and *DBC1* silencing

Expression of *DBC1* was analyzed in 25 bone marrow samples, including both methylated and unmethylated specimens. RNA was isolated using the MagNa Pure LC mRNA HS kit, automated on the MagNa Pure robot (Roche Diagnostics GmbH, Mannheim, Germany). Total RNA was reverse-transcribed using random hexamer primers with the TaqMan® Gold RT-PCR Kit (Applied Biosystems). Quantitative estimation of the relative *DBC1* mRNA levels was performed by the ABI PRISM 7300 Sequence Detection Instrument and software (Applied Biosystems) with specific oligonucleotides and pre-developed TaqMan Assays (Assay-on-Demand®, Applied Biosystems) following the manufacturer's protocol. The relative quantification of *DBC1* levels was expressed as follows: 2^−[^ 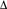 ^*C*t(AML samples)−^ 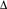 ^*C*t(CD34+cells)]^ = 2^−^ 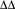 ^*C*t^, where *ΔC*_t_ = *C*_t_(DBC1)−*C*_t_(*GAPDH*) [22]. MSP-positive and MSP-negative cases were compared using the Mann-Whitney U test.

## Results

### Methylation profiling of AML

**Figure 1:**
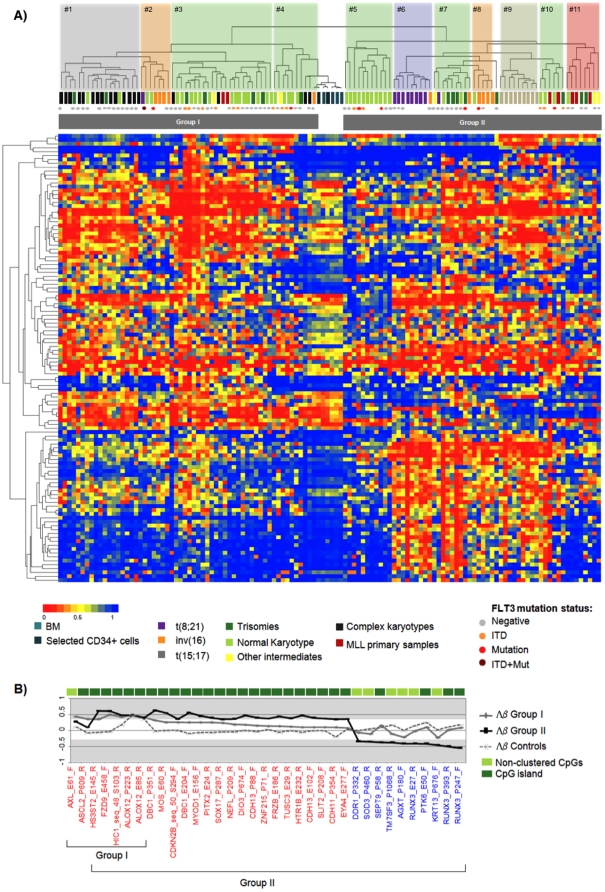
Unsupervised Clustering analysis of AML series and differential methylation status at specific CpG islands. A) Thumbnail overview of the two-way (probes against samples) hierarchical cluster obtained using the complete linkage method and correlation-based distance metric on 116 AML samples and 6 controls (columns) against 115 probes with variable β values (rows). β values are depicted using a pseudocolor scale. Samples are color-coded according to the prognostically relevant cytogenetic groups, determined on the basis of conventional chromosome-banding and fluorescence in situ hybridization analysis. Cluster numbers and methylation groups are indicated. The FLT3 status of the cases is shown. B) Graphical view of the of 35 selected differentially methylated CpG (red) and differentially unmethylated CpG (blue) loci in primary AML samples, along with the Δβ values of the control samples relative to 5 CD34+ selections obtained from cord blood samples. The areas corresponding to a Δβ>0.34 and <−0.34 are shaded. The significant probes for each group of samples are indicated.

DNA methylation levels were measured in 116 diagnostic AML samples (Table 1) and 6 control samples (see Material and Methods) at 1410 loci across the genome. Only the 115 CpGs with a standard deviation across all samples and controls >0.25 were selected for an unsupervised hierarchical cluster analysis that segregated the AML cohort into two main groups (Figure 1A). No statistically significant association was observed between the methylation groups and age, sex, F.A.B subtype or FLT3 status variables (Table 1). These two methylation groups showed a statistically significant association with specific prognostic cytogenetic groups (p<0.001, Fisher-Freeman-Halton exact test) (Table 1). Group I (58 patients) segregated 80% of the cases from the adverse cytogenetic prognostic group (cluster 1), 52% of cases from the intermediate cytogenetic prognostic group (clusters 3 and 4), 45% of cases with an inv(16) (cluster 2). Group II, with the same number of cases (n = 58), was distributed into 6 distinct smaller clusters, including those associated with chromosomal rearrangements t(8;21) (cluster 6), t(15;17) (cluster 9), inv(16) (cluster 8), MLL rearrangements (clusters 11), or normal Karyotypes (cluster 5) (Figure 1A). The analysis of the distribution of each of the eleven clusters identified showed for Karyotype, Cytogenetic Group, and F.A.B. subgroups, a very strong evidence of an unequal distribution among clusters (p-values for all three variables = 2*10^−6^, the smallest possible p-value attainable with 500000 simulations). For FLT3, a moderate evidence of unequal distribution (p-value = 0.02) was found. The distribution of the AML primary cases and the epigenetic signature for each cluster are shown on Tables S1 and S2.

Next, to gain further insight into the importance of these two main epigenetic signatures, we performed a differential methylation analysis (see Material and Methods section) to select. among the 115 probes used for hierarchical clustering, those 35 CpGs that were differentially methylated (Δß>0,34 & FDR<0.05, t-test) or unmethylated (Δß<−0,34 & FDR<0.05, t-test). We reasoned that these CpG sites represented those probes with changes on the methylation status in a large proportion of patients (concordantly altered probes) (Table S3). Focussing in those 35 selected CpGs, we observed that, while Group I showed only 7 differentially methylated CpGs, Group II included 23 differentially methylated CpGs (located at CpG islands and affecting to 20 genes,) and 10 differentially unmethylated CpGs (six of which were located at non-clustered CpGs) (Figure 1B). These results indicated aberrant promoter methylation of CpG islands and hypomethylation of selected non-clustered CpGs in the Group II AML cases.

The aberrantly methylated genes in Group II included tumor suppressor genes (*HIC1*), genes involved in cell cycle control (*DBC1*, *CDH13*, *CDKN2B*, *MOS*, and *PITX2*), development/differentiation (*CDH11*, *MYOD1*, *SOX17*), and apoptosis (*ALOX12*, *NEFL*). Interestingly, canonical pathway analysis of the 20 aberrantly methylated genes in AML Group II showed significant involvement of the Wnt/βcatenin signaling pathway through the aberrant methylation of *FRZB*, *FZD9*, and *SOX17* (p = 0.001, Fisher exact test & ratio 3/165). These 20 genes were distributed along chromosomes 2, 4, 6, 7, 8, 9, 11, 14, 16, and 17. Of the total number of probes on chromosomes 8 and 16 in the array, more than 10% were represented among our candidate genes. Kaplan-Meier plots did not reveal significant differences in the overall 5-year survival between AML Groups I and II (Figure S1).

### Methylation status of *DBC1* as a predictor of AML outcome in cases with a normal karyotype at diagnosis

**Figure 2:**
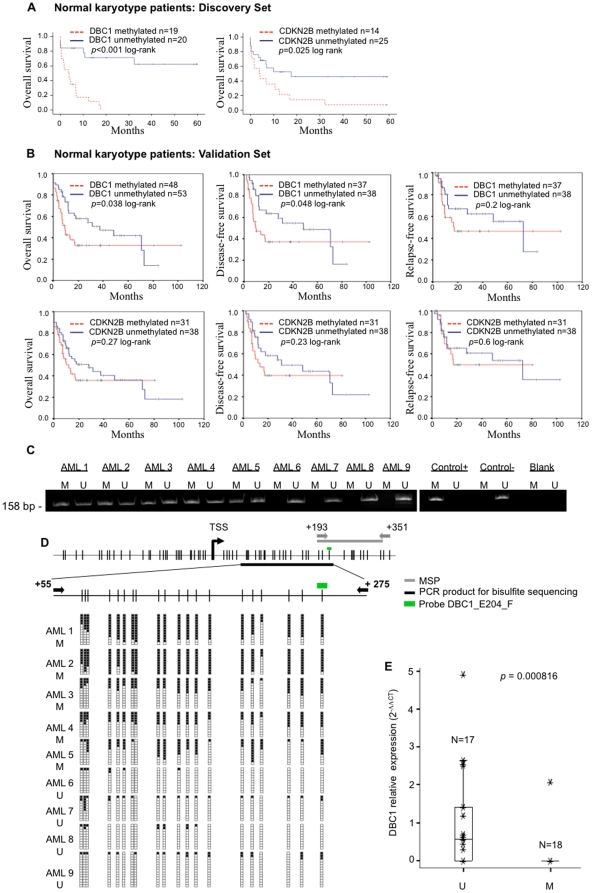
The methylation status of DBC1, influence in survival parameters and sequencing validation. A) Kaplan-Meier plots for the 39 patients with available clinical data from the original series and a normal karyotype at diagnosis, stratified by β values at the DBC1_E204_F and CDKN2_seq50 probes (a β value >0.5 was considered as positively methylated). B) Kaplan-Meier plots and DFS and RFS curves of the validation series, considering only patients with a normal karyotype and available clinical data, stratified by the methylation status (analyzed by MSP) of the *DBC1* and *CDKN2B* promoter. C) Examples of the MSP analysis of the *DBC1* gene. A visible PCR product in lane U indicates the presence of unmethylated *DBC1*; a visible product in lane M indicates the presence of methylated *DBC1*. CpGenome Universal Methylated DNA (Intergen, New York, NY, USA) was used as a positive control for methylated alleles. DNA from bone marrow donors was used as a negative control for methylated genes. Water controls for the PCR reaction are also shown. Samples AML1, AML2, AML3, AML4, and AML5 were methylation-positive, whereas all the others were methylation-negative. D) Status of 20 CpGs in the *DBC1* gene assessed by bisulfite genomic sequencing analysis on 9 AML samples. Primer design for MSP (black arrows) and bisulfite sequencing (gray arrows) is indicated. The green bar above the diagram of the *DBC1* CpG island indicates the location of the probe used in the methylation arrays. Black squares, methylated CpG; white squares, unmethylated CpG dinucleotides. E) Box plot of the *DBC1* relative transcript expression measured by quantitative PCR. MSP-positive and negative cases were compared using the Mann-Whitney U test.

To determine the prognostic value of the methylation status of specific genes, we used the bioinformatic tool SignS, with the ß values of the same 115 CpGs that showed a larger variation across the original series (same as above). When the cases with a normal karyotype (n = 39) were studied, a significant lower overall survival rate was observed among the patients with aberrant methylation of two CpGs, DBC1_E204_F and CDKN2B_sec50 (Figure S2). Conventional Kaplan-Meier analysis confirmed these results (Figure 2A).

The selected *DBC1* and *CDKN2B* CpGs were studied using MSP in an independent series of 244 and 151 cases, respectively. Similar frequencies and distributions to those of the original series were observed (Table 2). In cases with a normal karyotype, the presence of a positive methylated allele within the *CDKN2B* promoter was not found to be significantly associated with poor outcome in the validation cohort. In contrast, aberrant promoter methylation of *DBC1* was found to be associated with a significantly worse ten-year survival (overall survival, p = 0.038 and disease-free survival, p = 0.048 by the log-rank test). The estimated median overall survival was 57 months for the unmethylated group compared with 15 months for the methylated group. No differences were observed in relapse rates between patients in the methylated or unmethylated groups (Figures 2B and 2C). Furthermore, a statistically significant association was not observed between aberrant methylation of *DBC1* and other important known prognostic factors in AML harboring a normal karyotype, such as *NPM1* mutations, *FLT3* mutational status, and response to treatment. An even distribution of cases with aberrantly methylated *DBC1* was observed for *NPM1* mutations and treatment response (Table 3). Interestingly, *FLT3* aberrations were two times more prevalent in the methylated *DBC1* group and the presence of both hits was associated with a significantly worse ten-year overall survival (overall survival, p = 0.0075 and disease-free survival, p = 0.0071 by the log-rank test) (Figure S3).

**Table 2:** Distribution of aberrant methylation of *DBC1* and *CDKN2B* promoters in two independent AML series.

	DBC1*						CDKN2B*					
	Original Set Illumina Array			Validation Set MSP Analysis			Original Set Illumina Array			Validation Set MSP Analysis		
	N	Methylated		N	Methylated		N	Methylated		N	Methylated	
**Favorable Group**												
t(15;17)	9	9	(100%)	19	16	(84%)	9	5	(55.5%)	7	3	(42%)
inv(16)	11	9	(82%)	9	5	(56%)	11	4	(36.4%)	0	-	
t(8;21)	10	10	(100%)	12	8	(66%)	10	7	(70%)	3	2	(66%)
**Intermediate Group**												
Normal Karyotype	41	21	(51.2%)	111	54	(49%)	41	15	(36.6%)	99	42	(42%)
Trisomy 8	7	6	(86%)	12	6	(50%)	7	2	(28.6%)	3	1	(33%)
Other Intermediate	13	9	(69%)	21	12	(52%)	13	4	(31%)	10	3	(30%)
**Adverse Group**												
Complex Karyotype	14	1	(7%)**	16	11	(69%)	14	3	(21.4%)	7	2	(29%)
MLL	7	7	(100%)***	2	0	(0%)	7	1	(14%)	2	1	(50%)
Other Adverse	4	1	(25%)	7	1	(14%)	4	1	(25%)	2	1	(50%)
Other	-		-	35	15	(42%)	-	-		18	6	(33%)
Total	116	71	(61%)	244	128	(52%)	116	43	(37%)	151	61	(40%)

MLL, mixed lineage leukemia; MSP, methylation-specific polymerase chain reaction; N, number of cases. (*)The frequencies of methylated *DBC1* and *CDKN2B* in both sets of samples were compared in all categories using the Fisher exact test. There were no statistical differences in the frequencies, except for (**) p = 0.001 and (***) p = 0.28.

Other: Cases with no available cytogenetic data.

**Table 3:** Distribution of *DBC1* promoter methylation among patients with a normal karyotype at diagnosis.

	Discovery Set							
	GoldenGate Methylation				Validation Set			
	Cancer Panel I				MSP Analysis			
	N	UNMET	MET	p value#	N	UNMET	MET	p value#
**FLT3-ITD**	38	4/18∗	8/20	NS	90	7/44∗	14/46∗	NS
***NPM1***	-	-	-	-	67	15/33∗	16/34∗	NS
**Gender**	39			NS	111			NS
• Male		8/19	9/20			28/57	26/54	
• Female		11/19	11/20			29/57	28/54	
**Age**	39			NS	111			NS
• <60 years-old		4/19	9/20			37/57	29/54	
• >60 years-old		15/19	11/20			20/57	25/54	
**WBC**	39			NS	111			NS
• <10×10^9^/L		6/19	7/20			28/57	28/54	
• >10×10^9^/L		13/19	13/20			29/57	26/54	
**FAB Sub-type**	39			NS	111			NS
• M0		0/19	1/20			1/57	5/54	
• M1		5/19	5/20			16/57	14/54	
• M2		5/19	4/20			16/57	15/54	
• M3		0/19	0/20			0/57	0/54	
• M4		6/19	3/20			13/57	10/54	
• M5		3/19	7/20			7/57	4/54	
• M6		0/19	0/20			1/57	3/54	
• M7		0/19	0/20			0/57	1/54	
• Unclassified		0/19	0/20			3/57	2/54	
**Treatment Response**		-		-	101			NS
• Complete Remission	-	-	-			38/48	37/53	
• Resistance		-	-			6/48	9/53	
• Death		-	-			4/48	7/53	

MSP, methylation-specific polymerase chain reaction; NS, non-significant; MET, methylated; UNMET, unmethylated.

# p values were calculated using Fisher's exact test. p<0.05 was considered statistically significant.

∗The mutational status of *FLT3* and *NPM1* was not available for all patients.

Multivariate proportional-hazard analysis revealed that *DBC1* methylation status was not independent of other risk factors determined to be significant in the model, such as *FLT3* mutations, age, and response to treatment (Table S4).

Finally, we determined the extent of aberrant methylation of *DBC1* by bisulfite sequencing of the genomic region where the DBC1_E204_F was located. Nine patient's samples, with a known methylation status assessed by MSP (positive and negative) at the *DBC1* promoter, were further studied. No densely methylated CpGs were found in the MSP-negative samples (n = 4), whereas all the analyzed MSP-positive cases (n = 5) were found to have a median of 16 positive clones in the 20 CpGs analyzed (Figure 2D). Furthermore, we found that aberrant methylation of *DBC1* was associated with reduced expression, as measured by quantitative PCR. Complete *DBC1* silencing was observed in over 90% (17 out of 18) of the MSP-positive cases and in 35% (6 out of 17) of the MSP-negative samples (Figure 2E).

### Effect of fusion proteins on the epigenetic profiling of AML

The methylation profile of primary AML samples at diagnosis suggested a direct interaction between fusion proteins and DNA methylation (Figure 1A). To investigate the role of fusion proteins in the methylation signature, we studied 20 HSPC samples expressing the *MLL/AF9* (HSPC-MA9) or the core binding factor (CBF) fusion proteins *AML1/ETO* or *CBFB/MYH11* (HSPC-AE or HSPC-CM), which have been characterized elsewhere [13].

**Figure 3:**
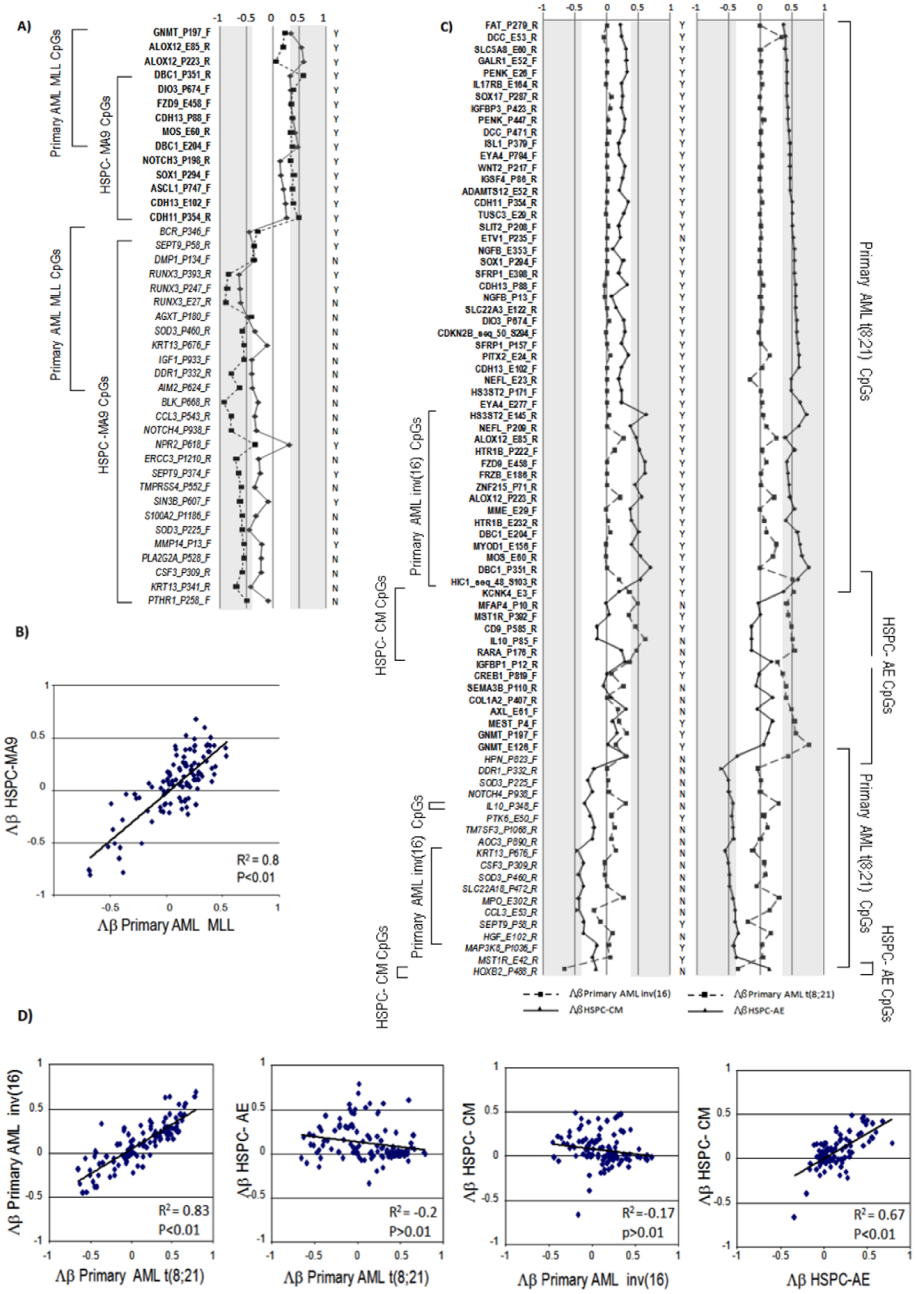
Comparison of the methylation patterns of primary samples and hematopoietic stem cells/progenitor cells. A) Graphical view of the 41 selected differentially methylated CpGs (bold) and differentially unmethylated CpGs (italics) in primary MLL AML samples and in the AML HSPC-MA9 samples. The area corresponding to a Δβ>0.34 or <−0.34 is shaded. The figure shows whether the selected CpG is included (Y) or not (N) in a CpG island. B) Scatter plot of the Δβ values of the 115 CpGs selected to define the AML methylation signatures. C) Graphical view of the 81 selected differentially methylated CpGs (bold) and differentially unmethylated CpGs (italics) in primary AML samples harboring t(8;21) or inv(16) and in the HSPC-AE and HSPC-CM samples. The areas corresponding to a Δβ>0.34 and <−0.34 are shaded. The figure shows whether the selected CpG is included (Y) or not (N) in a CpG island. D) Scatter plots of the Δβ values of the 115 CpGs selected to define the AML methylation signatures.

To define the *MLL* methylation signature, we selected 144 CpGs that showed an standard deviation across MLL and control samples >0.25 (Table S5). Unsupervised hierarchical clustering revealed that primary MLL cases and HSPC-MA9 samples were easily separated from the healthy control samples, and distinct methylation signatures were obtained for MLL samples (Figures S4A and S5B). Forty-one CpGs were differentially methylated between AML and the control samples (Table S5). Six out of nine differentially methylated CpGs (all within CpG islands) involving the *DBC1*, *DIO3*, *FZD9*, *CDH13*, and *MOS* genes, and 11 out of 12 differentially unmethylated CpGs (70% non-clustered CpGs) identified in primary MLL showed the same status in the HSPC-MA9 samples (Figure 3A and Table S5). Furthermore, a statistically significant positive correlation between primary MLL and myeloid HSPC-MA9 samples was observed when comparing the Δß values at the 115 CpGs with a larger variation across the original series (Figure 3B). These results indicated that the *MLL/AF9* fusion protein drives the methylation signature harbored by the primary AML MLL cases.

To establish the role of the lymphoid/myeloid lineage commitment in the methylation signature, we analyzed a set of 13 primary ALL samples. Unsupervised hierarchical clustering revealed that primary MLL and HSPC-MA9 samples had a methylation signature that was different from that of primary ALL samples harboring the *TEL/AML1* or *BCR/ABL* fusion genes (Figure S4B). Five times more differentially methylated CpGs across the genome were observed in the lymphoid MLL than in the myeloid MLL samples (Table S5) and the *DBC1*, *DIO3*, *FZD9*, and *MOS* genes were identified as differentially methylated CpGs in all the MLL samples. These results suggest an important contribution of DNA methylation to cell lineage commitment with a non-random epigenomic signature in MLL.

To determine the CBF methylation signature, we selected 110 CpGs with an standard deviation across CBF samples and controls >0.25. Unsupervised hierarchical clustering revealed that HSPC-CBF samples segregated with the healthy control samples (Figure S5A), and we did not identify a common signature between primary samples and HSPC-CBF samples. Among the 81 CpGs selected as differentially methylated (Table S6), primary CBF cases showed two to three times more differentially methylated CpGs than HSPC-CBF samples, and over 95% of the differentially methylated CpGs on the primary CBF cases were located at CpG islands compared with only half of the differentially methylated CpGs selected for the HSPC-CBF samples (Figure 3C). Furthermore, the CBF methylation signature that includes the 15 differentially methylated CpGs and 9 differentially unmethylated CpGs identified on primary CBF samples was not present in HSPC-CBF samples (Table S6). Finally, when the Δß values at the 115 CpGs were compared with larger variations across the original series, a significant positive correlation (R^2^>0.83) between the t(8;21) and inv(16) primary AML patients and an absence of correlation between primary samples and their respective HSPC models was observed (Figure 3D). These data confirmed the presence of a partially common methylation signature between cases of primary CBF leukemia and demonstrated that these fusion proteins alone are not capable of recapitulating the methylation signature observed in their respective primary AML samples.

## Discussion

The present report is a systematic study of DNA methylation patterns on adult *de novo* AML. Our unsupervised clustering analysis of 116 patients revealed that distinct epigenetic signatures could be identified based on a limited number of genes. Recent reports, using different epigenomic approaches with a much larger number of CpGs [8], [9], also defined epigenetic profiles for cases harboring balanced translocations such as t(8;21), inv(16), t(15;17), or MLL rearrangements. Nevertheless, our data showed that most of these cases with chromosome rearrangements were clustered in a large group (Group II) that shows abundant and common epigenetic modifications on CpG islands and hypomethylation of selected non-clustered CpGs. Within Group II, we identified a subset of 20 genes conforming an epigenetic signature common to AML with fusion proteins and a subset of AML cases from the intermediate cytogenetic prognostic subgroup. Furthermore, the pathway analysis of these aberrantly methylated genes showed a significant involvement of the Wnt signaling pathway. These results are consistent with our recent studies and reveal a significant role of the epigenetic modifications of Wnt antagonists in the pathogenesis of AML [23], [24]. On the other hand, cases harboring complex karyotypes clustered within Group I AML, which did not show a hypermethylated signature, supporting the hypothesis put forward by Koeger et al. of a negative correlation between epigenetic and chromosomal instability [7].

Our results, along with previous studies, suggested a link between genetic/chromosome rearrangements and the induction of aberrant DNA methylation [8], [9], [11], [25]. We previously showed that the overexpression of the MLL/AF9 fusion protein on HSPC induced acute myeloid, lymphoid, or mixed-lineage leukemia in immunodeficient mice, and that these transformed HSPC could be lineage-directed by altering either the growth factors or the recipient mouse strain [13]. Using this leukemic HSPC model, we demonstrated that *MLL/AF9* recapitulated the epigenetic profile observed in the MLL-positive patients, as has been shown in mice models with other genetic insults [25], thus supporting a direct role for MLL fusion proteins in the down-regulation of target genes by DNA methylation [26]. However, the mechanisms underlying this signature have yet to be explored. Furthermore, the observed ALL MLL methylation profile included a significantly higher number of aberrantly methylated CpGs than the AML cases. These results support the essential role of DNA methylation in the plasticity of the hematopoietic system [27], [28], suggesting interplay between transcription factors downstream of cytokine receptors and the DNA methylation machinery.

Conversely, *AML1/ETO* and *CBPβ/MYH11* overexpression on HSPC failed to reproduce the epigenetic signature observed in the primary patients, suggesting that these two CBF fusion proteins are insufficient to target DNA methylation to specific sites, as they are not capable of inducing a fully transformed phenotype [29], [30]. These results suggest that, either longer periods of time (more than 12 weeks) are needed to induce the epigenetic modifications or that these fusion proteins do not have a direct impact on the DNA methylation profile (as is shown to happen in the HSPC model). Further research exploring the mechanisms driving the specific epigenetic signature of the different cytogenetic subgroups seems warranted.

In addition, another major objective of our study was to determine whether patterns of DNA methylation signature could be used to improve patient prognostification, mainly among the abundant normal karyotype group of cases. As other [8] we were not able to correlate the presence of a methylation signature with different clinical outcomes among normal karyotype cases, as it has been previously reported using larger number of CpGs [9]. However, using specific biomarkers, differences in overall survival in patients with AML have been revealed by analysis of concurrent aberrant methylation of specific genes, such as *ESR1*, *CDKN2B*/*p15*, and *IGSF4* [31]. Using the SignS web tool, we were able to identify the methylation status of the deleted in bladder cancer 1 (*DBC1*) and the cyclin-dependent kinase inhibitor 2B (*CDKN2B/p15*) as predictors of overall survival in the AML subgroup with a normal karyotype. Only aberrant methylation of the *DBC1* promoter was observed to have statistically significant prognostic value among cases with a normal karyotype in an independent case series. *DBC1* and *CDKN2B* have been shown to have tumor-suppressor activity through the negative regulation of G1 cell cycle progression, and their loss of function has been associated with an advantage in proliferation, growth, and malignant transformation [32], [33]. Our results suggest that the clinical importance of *CDKN2B* methylation in AML is still controversial [34].

Hypermethylation associated with reversible epigenetic silencing of *DBC1* has been reported in hematological disorders [19], [35], [36] and in solid tumors [37], [38]. This silencing mechanism has been postulated as an early and age-independent event in the development of malignancy [35], [38]. We identified aberrant methylation of *DBC1*, located at 9q33.1, as an adverse prognostic marker in the AML subgroup with a normal karyotype. Furthermore, when this epigenetic event is combined with *FLT3* status, it constitutes a unique and powerful predictor of clinical outcome within AML cases with a normal karyotype. However, under current therapeutic regimens, this epigenetic marker does not retain its independence in the multivariate analysis. The identification of patients with aberrant DNA methylation patterns that can predict survival will be essential in future designs of clinical trials with demethylating agents.

In conclusion, comprehensive epigenetic profiling of AML provides relevant biological information and new clinical markers that should be integrated in the design of clinical trials with demethylating agents.

## Supporting Information

Document S1 PCR primers and conditions
(0.06 MB DOC)

Table S1 Clinical & Genetics of the cases included on each cluster
(0.09 MB DOC)

Table S2 Methylation values for the statistically significant probes
(0.07 MB XLS)

Table S3 Methylation status of 35 CpG loci selected as differentially methylated between the AML and BM control group.
(0.13 MB DOC)

Table S4 Multivariate Cox regression from treated patients with a normal karyotype at diagnosis.
(0.19 MB DOC)

Table S5 Methylation status of 105 CpGs selected as differentially methylated between primary MLL cases or HSPC-MA9 samples and controls.
(0.26 MB DOC)

Table S6 Methylation status of 81 CpGs selected as differentially methylated between primary CBF leukemia cases or HSPC-CBF samples and controls.
(0.36 MB DOC)

Figure S1 Kaplan-Meier curves for overall survival in cases with available clinical data stratified by methylation signature: A) All cases; B) Patients with a normal karyotype.
(1.84 MB TIF)

Figure S2 A DNA Methylation Classifier to predict Clinical Outcome in the AML cases included on the intermediate cytogenetic subgroup or in the Normal karyotype cases. Results obtained using the Beta values of the 115 CpGs with a larger variation across the original series, the SignS Web tool for gene selection and signature finding, build a predictor model of overall survival based on the methylation status of two CpGs, DBC1_E204_F and CDKN2B_sec50. Survival curves comparing low-score and high-score models from final models, with those two probes, using boosting of a component-wise Cox model (A and B) and the threshold gradient descent method (B and C) are shown.
(3.01 MB TIF)

Figure S3 Kaplan-Meier overall survival and disease-free survival curves for patients with available clinical data and a normal karyotype at diagnosis from the validation series, stratified by MSP result and the DBC1 gene and the FLT3 status.
(2.76 MB TIF)

Figure S4 A) Unsupervised hierarchical clustering by applying the complete linkage method and uncentered based distance metric for 7 AML-primary MLL cases, 3 primary ALL-MLL cases, 10 HSPC-MA9 (5 myeloid and 5 lymphoid), and 11 controls (4 bone marrow, 2 selected CD34+, and 5 CB) in the 144 probes selected by filtering with a standard deviation over 0.25 across all samples. B) Unsupervised hierarchical clustering by applying the complete linkage method and uncentered based distance metric for 13 ALL primary cases (3 MLL cases, 5 TEL/AML1 cases, and 5 BCR/ABL cases), 5 lymphoid HSPC-MA9, and 11 controls (4 bone marrow, 2 selected CD34+ samples, and 5 cultured cord blood samples), using 51 probes with a standard deviation over 0.25 across all the samples.
(6.01 MB TIF)

Figure S5 A) Unsupervised hierarchical clustering with the complete linkage method and euclidean-based distance metric of the primary CBF leukemia cases, HSPC-CBF and controls (bone marrow and cord blood). B) Unsupervised hierarchical clustering of the primary AML MLL leukemia cases, myeloid HSPC-MA9 and controls (bone marrow and cord blood) performed with 90 selected probes after filtering with an SD>0.25.
(6.01 MB TIF)
