# Supplementary material for: DNA Methylation Profiles and Their Relationship with Cytogenetic Status in Adult Acute Myeloid Leukemia
Source: PLoS One. 2010 Aug 16;5(8):e12197. doi: 10.1371/journal.pone.0012197 (PMC2922373; doi:10.1371/journal.pone.0012197)
Supplement: Document S1 — PCR primers and conditions (0.06 MB DOC) [file pone.0012197.s001.doc]

PCR Conditions, primers and expected product sizes

| Bisulfite sequencing PCR | |  |  |  |
| --- | --- | --- | --- | --- |
| NAME | SEQUENCE |  | PRODUCT SIZE | PCR CONDITIONS |
| BS-DBC1v5-s | AYGGTTGTAAATTGATTTGG | | 220 bp |  |
| BS-DBC1v5-as | CCTAACTCCTAACAACCTAACTCA | |  |  |
|  |  |  |  |  |
| Methylation-specific PCR (MSP) |  |  |  |  |
| DBC1 |  |  |  |  |
| NAME | SEQUENCE | | PRODUCT SIZE | PCR CONDITIONS |
| DBC1-1 | 5'-TGTTTGTTTTCGGGATTAGC-3' | | 158 bp | 10 min at 95ºC |
| DBC1-2 | 5'-ACGCTTTTTATTCGACTCGA-3' | |  | 40 cycles of 30 sec at 95ºC, 60ºC and 72ºC |
| DBC1-3 | 5'-TTATGTTTGTTTTTGGGATTAGT-3' | |  | 7 min at 72ºC |
| DBC1-4 | 5'-AAAACACTTTTTATTCAACTCAA-3' | |  |  |
| CDKN2B |  |  |  |  |
| CDKN2B-1 | 5'-TTAGGAAGGAGAGAGTGCGTC-3' | | 132 bp | 15 min at 95ºC |
| CDKN2B-2 | 5'-TACCCTTATTCTCCTCGCG-3' | |  | 35 cycles of 30 sec at 95ºC, 45 sec at 60ºC |
| CDKN2B-3 | 5'-TTAGGAAGGAGAGAGTGTGTT-3' | |  | and 10 min at 72ºC |
| CDKN2B-4 | 5'-TACCCTTATTCTCCTCACA-3' | |  | 10 min at 72ºC |
|  |  |  |  |  |
| Quantitative PCR |  |  |  |  |
| DBC1 |  |  |  |  |
| NAME | ASSAY REFERENCE | | PRODUCT SIZE | PCR CONDITIONS |
| DBC1 | Hs01089684_m1 (junction between E2 and E3) | | 71 bp | 2 min at 50°C |
| GAPDH | Hs99999905_m1 | |  | 10 min at 95ºC |
|  |  | |  | 40 cycles of 15 sec at 95ºC and 1 min at 60ºC |
