# Supplementary material for: DNA Methylation Profiles and Their Relationship with Cytogenetic Status in Adult Acute Myeloid Leukemia
Source: PLoS One. 2010 Aug 16;5(8):e12197. doi: 10.1371/journal.pone.0012197 (PMC2922373; doi:10.1371/journal.pone.0012197)
Supplement: Table S1 — Clinical & Genetics of the cases included on each cluster (0.09 MB DOC) [file pone.0012197.s002.doc]

|  |  | Methylation Group I | | | | | |  | Methylation Group II | | | | | | |
| --- | --- | --- | --- | --- | --- | --- | --- | --- | --- | --- | --- | --- | --- | --- | --- |
| Cluster# |  | 1 | 2 | | 3 | | 4 |  | 5 | 6 | 7 | 8 | 9 | 10 | 11 |
| Nº of Included cases | | 18 | 7 | | 24 | | 9 |  | 11 | 9 | 9 | 5 | 9 | 7 | 8 |
| Cytogenetic |  | Adverse | Inv(16) | | Interm. | | Interm |  | Normal | t(8;21) | Interm. | Inv(16) | t(15;17) | Interm. | MLL |
| Hallmark |  | (77,7%) | (57,4%) | | (83.3%) | | .(67.7%) |  | Karyotype(100%) | (91%) | (77.7%) | (80%) | (88,8%) | (66.6%) | (60%) |
| **FAB Sub-type** |  |  |  | |  | |  |  |  |  |  |  |  |  |  |
| M0 | 4 | 2 |  | | 1 | |  |  |  |  | 1 |  |  |  |  |
| M1 | 19 | 6 |  | | 6 | | 1 |  | 3 |  | 2 |  |  |  | 1 |
| M2 | 30 | 4 | 1 | | 7 | | 4 |  | 1 | 8 | 3 | 1 |  | 1 |  |
| M3 | 9 |  |  | |  | |  |  |  |  |  |  | 8 | 1 |  |
| M4 | 13 | 3 | 1 | | 4 | | 0 |  | 2 |  |  |  |  | 2 | 1 |
| M4EO | 11 |  | 4 | | 0 | | 1 |  |  | 1 | 1 | 4 |  |  |  |
| M5 | 27 | 2 | 1 | | 4 | | 3 |  | 5 |  | 2 |  | 1 | 3 | 6 |
| M6 | 3 | 1 |  | | 2 | |  |  |  |  |  |  |  |  |  |
| **Cytogenetic Prognosis Group** | | |  | |  | |  |  |  |  |  |  |  |  |  |
| **Favorable** | **30** |  | **5** | |  | | **1** |  | **0** | **9** | **2** | **4** | **8** | **1** | **0** |
| t(8;21) | 10 |  | 1 | |  | |  |  | 0 | 8 | 1 |  |  |  |  |
| inv(16) | 11 |  | 4 | |  | | 1 |  | 0 | 1 | 1 | 4 |  |  |  |
| t(15;17) | 9 |  |  | |  | |  |  |  |  |  |  | 8 | 1 |  |
| **Intermediate** | **61** | **4** | **2** | | **20** | | **6** |  | **11** | **0** | **7** | **1** | **1** | **4** | **5** |
| Normal Karyotype | 41 | 2 | 2 | | 16 | | 3 |  | 11 |  | 2 | 1 |  | 3 | 1 |
| Single Trisomy | 12 | 1 |  | | 3 | | 1 |  |  |  | 4 |  | 1 | 1 | 1 |
| Double Trisomy | 3 | 1 |  | | 1 | |  |  |  |  |  |  |  |  | 1 |
| Other Intermediate | 5 |  |  | | 0 | | 2 |  |  |  | 1 |  |  |  | 2 |
| **Adverse** | **25** | **14** | **0** | | **4** | | **2** |  | **0** | **0** | **0** | **0** | **0** | **2** | **3** |
| Complex Karyotype | 14 | 11 |  | | 1 | | 2 |  |  |  |  |  |  |  |  |
| MLL | 7 |  |  | | 2 | |  |  |  |  |  |  |  | 2 | 3 |
| Other Adverse | 4 | 3 |  | | 1 | |  |  |  |  |  |  |  |  |  |
| **FLT3** |  |  |  | |  | |  |  |  |  |  |  |  |  |  |
| ITD | 15 |  |  | | 7 | | 2 |  | 3 |  |  |  |  | 3 |  |
| Mutation | 6 |  | 1 | |  | | 1 |  | 1 |  | 1 | 1 |  | 1 |  |
| ITD+Mutation | 1 |  | 1 | |  | |  |  |  |  |  |  |  |  |  |
| Negative | 61 | 14 | 1 | | 15 | | 5 |  | 7 | 4 | 7 | 1 | 1 |  | 6 |
| ND | 33 | 4 | | 4 | | 2 | 1 |  |  | 5 | 1 | 3 | 8 | 3 | 2 |
