# Supplementary material for: DNA Methylation Profiles and Their Relationship with Cytogenetic Status in Adult Acute Myeloid Leukemia
Source: PLoS One. 2010 Aug 16;5(8):e12197. doi: 10.1371/journal.pone.0012197 (PMC2922373; doi:10.1371/journal.pone.0012197)
Supplement: Table S3 — Methylation status of 35 CpG loci selected as differentially methylated between the AML and BM control group. (0.13 MB DOC) [file pone.0012197.s004.doc]

| ID PROBE | ^*^CpG island | GENE NAME | Chr. | AML PRIMARY SAMPLES | | | | | | | | CONTROLS | | | | | |
| --- | --- | --- | --- | --- | --- | --- | --- | --- | --- | --- | --- | --- | --- | --- | --- | --- | --- |
|  |  |  |  | Group I | | | | Group II | | | | BM controls | | CB controls | | BM vs CB | |
|  |  |  |  | (n=58) | | | | (n=58) | | | | (n=6) | | (n=5) | |  |  |
|  |  |  |  | Mean | STD | ^**^Δß | ^#^FDR | Mean | STD | ^**^Δß  | ^#^FDR | Mean | STD | Mean | STD | *** | ^#^FDR |
|  |  |  |  | β value |  |  |  | β value |  |  |  | β value |  | β value |  | Δß  |  |
|  |  |  |  |  |  |  |  |  |  |  |  |  |  |  |  |  |  |
| AGXT_P180_F | N | alanineglyoxylate aminotransferase | 2 | 0.71 | 0.27 | -0.22 | ***0.01*** | 0.52 | 0.29 | -0.41 | ***0.001*** | 0.93 | *0.04* | 0.93 | 0.04 | -0.01 | *NS* |
| AXL_E61_F | N | AXL receptor tyrosine kinase isoform 1 | 19 | 0.53 | 0.32 | 0.44 | ***0.01*** | 0.37 | 0.33 | 0.28 | ***0.022*** | 0.09 | *0.02* | 0.18 | *0.09* | 0.1 | *NS* |
| DDR1_P332_R | N | discoidin domain receptor family, member 1 isoform b | 6 | 0.76 | 0.20 | -0.07 | *NS* | 0.48 | 0.31 | -0.34 | ***0.005*** | 0.83 | *0.05* | 0.81 | *0.09* | -0.02 | *NS* |
| KRT13_P676_F | N | keratin 13 isoform b | 17 | 0.51 | 0.25 | -0.23 | ***0.02*** | 0.28 | 0.20 | -0.46 | ***0.000*** | 0.74 | *0.05* | 0.75 | *0.12* | 0 | *NS* |
| RUNX3_E27_R | N | runt-related transcription factor 3 isoform 1 | 1 | 0.77 | 0.21 | -0.01 | *NS* | 0.37 | 0.33 | -0.41 | ***0.006*** | 0.78 | *0.08* | 0.92 | *0.05* | 0.14 | *0.03* |
| SOD3_P460_R | N | superoxide dismutase 3, extracellular | 4 | 0.63 | 0.23 | -0.11 | *NS* | 0.38 | 0.29 | -0.35 | ***0.001*** | 0.74 | *0.04* | 0.77 | *0.13* | 0.03 | *NS* |
| TM7SF3_P1068_R | N | transmembrane 7 superfamily member 3 | 12 | 0.50 | 0.25 | -0.12 | *NS* | 0.25 | 0.20 | -0.38 | ***0.018*** | 0.62 | *0.18* | 0.78 | *0.14* | 0.16 | *NS* |
| *ALOX12_E85_R* | *Y* | *arachidonate 12lipoxygenase* | 17 | 0.76 | 0.27 | 0.41 | *0.05* | 0.74 | 0.30 | 0.40 | ***0.044*** | 0.35 | *0.18* | 0.70 | *0.31* | 0.35 | *NS* |
| *ALOX12_P223_R* | *Y* | *arachidonate 12lipoxygenase* | 17 | 0.76 | 0.24 | 0.50 | ***0.00*** | 0.74 | 0.27 | 0.47 | ***0.002*** | 0.27 | *0.15* | 0.72 | *0.29* | 0.46 | *NS* |
| ASCL2_P609_R | Y | achaetescute complex homologlike 2 | 11 | 0.51 | 0.31 | 0.36 | ***0.01*** | 0.24 | 0.24 | 0.09 | *NS* | 0.15 | *0.02* | 0.06 | *0.02* | -0.09 | ***0.01*** |
| CDH11_P354_R | Y | cadherin 11, type 2 preproprotein | 16 | 0.37 | 0.20 | 0.09 | *NS* | 0.64 | 0.29 | 0.35 | ***0.004*** | 0.29 | *0.06* | 0.07 | *0.02* | -0.22 | ***0.01*** |
| CDH13_E102_F | Y | cadherin 13 preproprotein | 16 | 0.16 | 0.14 | 0.10 | ***0.01*** | 0.44 | 0.30 | 0.39 | ***0.001*** | 0.05 | *0.01* | 0.04 | *0.01* | -0.02 | *0.06* |
| CDH13_P88_F | Y | cadherin 13 preproprotein | 16 | 0.31 | 0.23 | 0.15 | ***0.03*** | 0.56 | 0.30 | 0.40 | ***0.002*** | 0.17 | *0.02* | 0.12 | *0.06* | -0.05 | *NS* |
| CDKN2B_seq_50_S294_F | Y | cyclin-dependent kinase inhibitor 2B isoform 1 | 9 | 0.31 | 0.30 | 0.26 | ***0.01*** | 0.40 | 0.34 | 0.36 | ***0.004*** | 0.05 | *0.02* | 0.03 | *0.00* | -0.01 | *NS* |
| DBC1_E204_F | Y | deleted in bladder cancer 1 | 9 | 0.41 | 0.22 | 0.25 | ***0.01*** | 0.72 | 0.19 | 0.55 | ***0.000*** | 0.17 | *0.04* | 0.05 | *0.01* | -0.11 | ***0.01*** |
| DBC1_P351_R | Y | deleted in bladder cancer 1 | 9 | 0.37 | 0.27 | 0.32 | ***0.00*** | 0.68 | 0.29 | 0.63 | ***0.000*** | 0.05 | *0.01* | 0.02 | *0.00* | -0.03 | *0.02* |
| DIO3_P674_F | Y | deiodinase, iodothyronine, type III | 14 | 0.21 | 0.24 | 0.18 | ***0.01*** | 0.51 | 0.25 | 0.47 | ***0.000*** | 0.03 | *0.01* | 0.02 | *0.00* | -0.01 | ***0.01*** |
| EYA4_E277_F | Y | eyes absent 4 isoform a | 6 | 0.13 | 0.10 | 0.07 | ***0.01*** | 0.42 | 0.32 | 0.36 | ***0.001*** | 0.06 | *0.01* | 0.09 | *0.01* | 0.02 | ***0.03*** |
| FRZB_E186_R | Y | frizzledrelated protein | 2 | 0.23 | 0.21 | 0.13 | *NS* | 0.54 | 0.32 | 0.44 | ***0.001*** | 0.10 | *0.01* | 0.02 | *0.00* | -0.07 | ***0.01*** |
| FZD9_E458_F | Y | frizzled 9 | 7 | 0.60 | 0.27 | 0.50 | ***0.00*** | 0.70 | 0.25 | 0.60 | ***0.000*** | 0.10 | *0.01* | 0.05 | *0.01* | -0.05 | ***0.01*** |
| HIC1_seq_48_S103_R | Y | hypermethylated in cancer 1 | 17 | 0.67 | 0.32 | 0.43 | ***0.01*** | 0.72 | 0.31 | 0.48 | ***0.001*** | 0.24 | *0.06* | 0.40 | *0.47* | 0.16 | *NS* |
| HS3ST2_E145_R | Y | heparan sulfate D glucosaminyl 3Osulfotransferase 2 | 16 | 0.45 | 0.29 | 0.35 | ***0.01*** | 0.71 | 0.30 | 0.61 | ***0.000*** | 0.10 | *0.03* | 0.03 | *0.01* | -0.07 | ***0.02*** |
| HTR1B_E232_R | Y | 5 hydroxytryptamine (serotonin) receptor 1B | 6 | 0.20 | 0.19 | 0.10 | *NS* | 0.57 | 0.31 | 0.47 | ***0.001*** | 0.10 | *0.03* | 0.03 | *0.02* | -0.07 | ***0.01*** |
| MOS_E60_R | Y | vmos Moloney murine sarcoma viral oncogene homolog | 8 | 0.34 | 0.28 | 0.26 | ***0.01*** | 0.62 | 0.26 | 0.54 | ***0.000*** | 0.08 | *0.02* | 0.07 | *0.03* | -0.01 | *NS* |
| MYOD1_E156_F | Y | myogenic differentiation 1 | 11 | 0.36 | 0.23 | 0.25 | ***0.01*** | 0.57 | 0.28 | 0.46 | ***0.000*** | 0.11 | *0.01* | 0.03 | *0.01* | -0.08 | ***0.01*** |
| NEFL_P209_R | Y | neurofilament, light polypeptide 68kDa | 8 | 0.30 | 0.20 | 0.19 | ***0.01*** | 0.47 | 0.31 | 0.36 | ***0.005*** | 0.11 | *0.03* | 0.05 | *0.01* | -0.06 | ***0.01*** |
| PITX2_E24_R | Y | paired-like homeodomain transcription factor 2 isoform c | 4 | 0.38 | 0.27 | 0.25 | ***0.01*** | 0.52 | 0.31 | 0.39 | ***0.002*** | 0.14 | *0.03* | 0.06 | *0.02* | -0.08 | ***0.01*** |
| PTK6_E50_F | Y | PTK6 protein tyrosine kinase 6 | 20 | 0.47 | 0.22 | 0.09 | *NS* | 0.15 | 0.19 | -0.42 | ***0.000*** | 0.57 | *0.11* | 0.80 | *0.12* | 0.24 | ***0.04*** |
| RUNX3_P247_F | Y | runt-related transcription factor 3 isoform 1 | 1 | 0.64 | 0.29 | 0.08 | *NS* | 0.17 | 0.21 | -0.55 | ***0.000*** | 0.72 | *0.14* | 0.88 | *0.06* | 0.17 | *NS* |
| RUNX3_P393_R | Y | runt-related transcription factor 3 isoform 1 | 1 | 0.73 | 0.28 | 0.02 | *NS* | 0.25 | 0.24 | -0.50 | ***0.000*** | 0.75 | *0.12* | 0.85 | *0.08* | 0.1 | *NS* |
| SEPT9_P58_R | Y | septin 9 | 17 | 0.70 | 0.27 | 0.16 | *0.08* | 0.49 | 0.31 | -0.37 | ***0.005*** | 0.86 | *0.06* | 0.94 | *0.03* | 0.08 | ***0.05*** |
| SLIT2_P208_F | Y | slit homolog 2 | 4 | 0.15 | 0.14 | 0.10 | ***0.02*** | 0.42 | 0.33 | 0.37 | ***0.004*** | 0.05 | *0.01* | 0.03 | *0.00* | -0.02 | ***0.02*** |
| SOX17_P287_R | Y | SRYbox 17 | 8 | 0.41 | 0.26 | 0.24 | ***0.02*** | 0.52 | 0.29 | 0.35 | ***0.003*** | 0.17 | *0.04* | 0.11 | *0.05* | -0.06 | *NS* |
| TUSC3_E29_R | Y | tumor suppressor candidate 3 isoform b | 8 | 0.18 | 0.16 | 0.12 | ***0.01*** | 0.43 | 0.30 | 0.38 | ***0.001*** | 0.05 | *0.01* | 0.04 | *0.02* | -0.02 | *NS* |
| ZNF215_P71_R | Y | zinc finger protein 215 | 11 | 0.30 | 0.18 | 0.14 | ***0.01*** | 0.53 | 0.27 | 0.37 | ***0.001*** | 0.16 | *0.03* | 0.17 | *0.08* | 0.01 | *NS* |

*# Mean ß values of each selected CpG locus from the AML samples included in each methylation signature and controls were estimated and FDR was applied to correct p values from the t test. An FDR<0.05 was considered statistically significant.* ^**^*Indicates whether the selected CpG is included (Y) or not (N) in a CpG island.* ^**^Δ*ß=(Mean ß value, AML group) – (Mean ß value, BM controls).*

^***^Δ*ß=(Mean ß value, BM control) – (Mean ß value, CB controls). dUMCpGs are underlined*
