# Supplementary material for: DNA Methylation Profiles and Their Relationship with Cytogenetic Status in Adult Acute Myeloid Leukemia
Source: PLoS One. 2010 Aug 16;5(8):e12197. doi: 10.1371/journal.pone.0012197 (PMC2922373; doi:10.1371/journal.pone.0012197)
Supplement: Table S4 — Multivariate Cox regression from treated patients with a normal karyotype at diagnosis. (0.19 MB DOC) [file pone.0012197.s005.doc]

| VARIABLE | 10-year Overall Survival | | 10-year Disease-Free | | 10-year Relapse-Free | |
| --- | --- | --- | --- | --- | --- | --- |
|  | (OS) | | Survival (DFS) | | Survival (RFS) | |
|  | (n=83) | | (n=62) | | (n=61) | |
|  | HR | *P* | HR | *P* | HR | *P* |
| **FLT3_ITD** |  |  |  |  |  |  |
| - Positive | 3.86 | *<0.001* | 2.4 | *0.035* | - | *NS* |
| - Negative |  |  |  |  |  |  |
| **Age** |  |  |  |  |  |  |
| - >60 years old | 3.08 | *0.001* | 2.6 | *0.008* | - | *NS* |
| -<60 years old |  |  |  |  |  |  |
| **White cell count** |  |  |  |  |  |  |
| - >10 x10^9^/L | - | *NS* | - | *NS* | - | *NS* |
| - <10 x10^9^/L |  |  |  |  |  |  |
| ***DBC1*** |  |  |  |  |  |  |
| - MSP-positive | - | *NS* | - | *NS* | - | *NS* |
| - MSP-negative |  |  |  |  |  |  |
| ***CDNK2B*** |  |  |  |  |  |  |
| - MSP-positive | - | *NS* | - | *NS* | - | *NS* |
| - MSP-negative |  |  |  |  |  |  |
| **Treatment response** |  |  |  |  |  |  |
| - Complete Response | 3.44 | *<0.001* | - | *NS* | - | *NS* |
| - Partial or No Response |  |  |  |  |  |  |
