# Supplementary material for: DNA Methylation Profiles and Their Relationship with Cytogenetic Status in Adult Acute Myeloid Leukemia
Source: PLoS One. 2010 Aug 16;5(8):e12197. doi: 10.1371/journal.pone.0012197 (PMC2922373; doi:10.1371/journal.pone.0012197)
Supplement: Table S5 — Methylation status of 105 CpGs selected as differentially methylated between primary MLL cases or HSPC-MA9 samples and controls. (0.26 MB DOC) [file pone.0012197.s006.doc]

|  |  |  |  | Primary MLL AML | | Myeloid HSPC-MA9 | | | Primary MLL ALL | | Lymphoid HSPC-MA9 | | | CB | BM |  |  |
| --- | --- | --- | --- | --- | --- | --- | --- | --- | --- | --- | --- | --- | --- | --- | --- | --- | --- |
|  | PROBE | ^*^CpG | CHR | *Mean ß* | ^**^Δß | *Mean ß* | ^***^ Δß | *^#^* FDR | *Mean ß value* | ^**^Δß | *Mean ß* | ^***^ Δß | *^#^* FDR | *Mean ß* | *Mean ß* | ^****^ Δß | *^##^*FDR |
|  |  | island |  | *value* |  | *value* |  |  |  |  | *value* |  |  | *value* | *value* |  |  |
| 1 | CDH13_E102_F | Y | 16 | 0.30 | 0.25 | 0.43 | 0.39 | 0.0158 | 0.18 | 0.12 | 0.75 | 0.71 | 0.0000 | 004 | 0.05 | 0.02 | 0.047 |
| 2 | CDH11_P354_R | Y | 16 | 0.56 | 0.27 | 0.56 | 0.49 | 0.0003 | 0.50 | 0.21 | 0.87 | 0.80 | 0.0001 | 0.07 | 0.29 | 0.22 | 0.011 |
| 3 | ASCL1_P747_F | Y | 12 | 0.54 | 0.21 | 0.60 | 0.38 | 0.0005 | 0.65 | 0.33 | 0.69 | 0.47 | 0.0024 | 0.22 | 0.32 | 0.11 | 0.026 |
| 4 | NOTCH3_P198_R | Y | 19 | 0.30 | 0.15 | 0.55 | 0.34 | 0.0001 | 0.76 | 0.61 | 0.79 | 0.58 | 0.0001 | 0.21 | 0.15 | -0.06 | NS |
| 5 | DBC1_P351_R | Y | 9 | 0.38 | 0.34 | 0.59 | 0.58 | 0.0002 | 0.92 | 0.88 | 0.84 | 0.83 | 0.0001 | 0.02 | 0.05 | 0.03 | 0.016 |
| 6 | GNMT_P197_F | Y | 6 | 0.48 | 0.35 | 0.58 | 0.24 | 0.0212 | 0.71 | 0.58 | 0.66 | 0.32 | 0.0025 | 0.35 | 0.14 | -0.21 | 0.076 |
| 7 | DIO3_P674_F | Y | 14 | 0.38 | 0.35 | 0.41 | 0.39 | 0.0366 | 0.42 | 0.39 | 0.68 | 0.66 | 0.0002 | 0.02 | 0.03 | 0.01 | 0.011 |
| 8 | FZD9_E458_F | Y | 7 | 0.45 | 0.35 | 0.41 | 0.36 | 0.0001 | 0.74 | 0.64 | 0.74 | 0.68 | 0.0001 | 0.05 | 0.10 | 0.05 | 0.011 |
| 9 | CDH13_P88_F | Y | 16 | 0.55 | 0.38 | 0.49 | 0.38 | 0.0050 | 0.30 | 0.13 | 0.70 | 0.58 | 0.0004 | 0.12 | 0.17 | 0.05 | NS |
| 10 | MOS_E60_R | Y | 8 | 0.51 | 0.43 | 0.42 | 0.35 | 0.0001 | 0.88 | 0.80 | 0.87 | 0.80 | 0.0001 | 0.07 | 0.08 | 0.01 | NS |
| 11 | DBC1_E204_F | Y | 9 | 0.65 | 0.48 | 0.42 | 0.37 | 0.0001 | 0.76 | 0.60 | 0.78 | 0.72 | 0.0001 | 0.05 | 0.17 | 0.11 | 0.011 |
| 12 | ALOX12_E85_R | Y | 17 | 0.90 | 0.55 | 0.90 | 0.20 | 0.0012 | 0.91 | 0.56 | 0.95 | 0.25 | 0.0095 | 0.70 | 0.35 | -0.35 | NS |
| 13 | ALOX12_P223_R | Y | 17 | 0.86 | 0.59 | 0.79 | 0.06 | 0.0169 | 0.61 | 0.34 | 0.83 | 0.10 | NS | 0.72 | 0.27 | -0.46 | NS |
| 14 | SOX1_P294_F | Y | 13 | 0.23 | 0.16 | 0.44 | 0.40 | 0.0259 | 0.82 | 0.74 | 0.69 | 0.65 | 0.0001 | 0.04 | 0.08 | 0.04 | 0.011 |
| 15 | BLK_P668_R | N | 8 | 0.64 | -0.27 | 0.02 | -0.92 | 0.0001 | 0.14 | -0.77 | 0.02 | -0.92 | 0.0000 | 0.94 | 0.91 | -0.04 | NS |
| 16 | RUNX3_E27_R | N | 1 | 0.18 | -0.60 | 0.03 | -0.89 | 0.0231 | 0.07 | -0.71 | 0.32 | -0.60 | 0.0002 | 0.92 | 0.78 | -0.14 | 0.03 |
| 17 | RUNX3_P393_R | Y | 1 | 0.12 | -0.63 | 0.02 | -0.83 | 0.0001 | 0.05 | -0.70 | 0.02 | -0.84 | 0.0000 | 0.85 | 0.75 | -0.10 | NS |
| 18 | RUNX3_P247_F | Y | 1 | 0.11 | -0.61 | 0.03 | -0.86 | 0.0001 | 0.04 | -0.68 | 0.02 | -0.86 | 0.0000 | 0.88 | 0.72 | -0.17 | NS |
| 19 | NOTCH4_P938_F | N | 6 | 0.49 | -0.30 | 0.08 | -0.78 | 0.0146 | 0.15 | -0.63 | 0.46 | -0.40 | 0.0005 | 0.86 | 0.78 | -0.08 | NS |
| 20 | DDR1_P332_R | N | 6 | 0.44 | -0.39 | 0.03 | -0.78 | 0.0296 | 0.23 | -0.60 | 0.24 | -0.57 | 0.0003 | 0.81 | 0.83 | 0.02 | NS |
| 21 | KRT13_P676_F | N | 17 | 0.33 | -0.41 | 0.05 | -0.69 | 0.0001 | 0.16 | -0.59 | 0.13 | -0.61 | 0.0001 | 0.75 | 0.74 | 0.00 | NS |
| 22 | CCL3_P543_R | N | 17 | 0.63 | -0.33 | 0.14 | -0.79 | 0.0001 | 0.42 | -0.55 | 0.54 | -0.39 | 0.0002 | 0.93 | 0.97 | 0.03 | 0.02 |
| 23 | S100A2_P1186_F | N | 1 | 0.59 | -0.31 | 0.25 | -0.58 | 0.0017 | 0.47 | -0.44 | 0.78 | -0.05 | - | 0.83 | 0.91 | -0.08 | 0.004 |
| 24 | CSF3_P309_R | N | 17 | 0.45 | -0.21 | 0.17 | -0.57 | 0.0001 | 0.22 | -0.44 | 0.71 | -0.02 | - | 0.74 | 0.66 | -0.07 | NS |
| 25 | MMP14_P13_F | Y | 14 | 0.24 | -0.20 | 0.27 | -0.54 | 0.0031 | 0.08 | -0.36 | 0.63 | -0.18 | 0.0209 | 0.82 | 0.43 | 0.38 | 0.035 |
| 26 | IGF1_P933_F | N | 12 | 0.18 | -0.39 | 0.04 | -0.54 | 0.0003 | 0.24 | -0.33 | 0.54 | -0.04 | - | 0.57 | 0.57 | 0.00 | NS |
| 27 | AIM2_P624_F | N | 1 | 0.39 | -0.38 | 0.19 | -0.63 | 0.0013 | 0.47 | -0.30 | 0.92 | 0.10 | - | 0.82 | 0.76 | -0.06 | NS |
| 28 | SEPT9_P58_R | Y | 17 | 0.51 | -0.35 | 0.58 | -0.35 | 0.0454 | 0.56 | -0.30 | 0.93 | -0.01 | - | 0.94 | 0.86 | -0.08 | 0.0412 |
| 29 | SEPT9_P374_F | Y | 17 | 0.19 | -0.23 | 0.08 | -0.64 | 0.0004 | 0.21 | -0.21 | 0.66 | -0.05 | 0.0367 | 0.72 | 0.42 | -0.30 | 0.0440 |
| 30 | TMPRSS4_P552_F | N | 11 | 0.49 | -0.33 | 0.34 | -0.59 | 0.0001 | 0.63 | -0.19 | 0.83 | -0.10 | - | 0.93 | 0.82 | 0.11 | 0.0097 |
| 31 | NPR2_P618_F | Y | 9 | 0.53 | 0.32 | 0.27 | -0.34 | 0.0140 | 0.14 | -0.07 | 0.46 | -0.15 | NS | 0.61 | 0.21 | -0.40 | NS |

| 32 | DMP1_P134_F | N | 4 | 0.55 | -0.34 | 0.35 | -0.36 | 0.0065 | 0.86 | -0.03 | 0.73 | 0.02 | - | 0.71 | 0.89 | 0.18 | 0.0164 |
| --- | --- | --- | --- | --- | --- | --- | --- | --- | --- | --- | --- | --- | --- | --- | --- | --- | --- |
| 33 | AGXT_P180_F | N | 2 | 0.46 | -0.47 | 0.53 | -0.40 | 0.0014 | 0.90 | -0.03 | 0.93 | 0.00 | - | 0.93 | 0.93 | 0.01 | NS |
| 34 | SOD3_P225_F | N | 4 | 0.54 | -0.33 | 0.35 | -0.58 | 0.0050 | 0.87 | 0.00 | 0.92 | -0.01 | - | 0.93 | 0.87 | -0.06 | NS |
| 35 | KRT13_P341_R | N | 17 | 0.83 | -0.10 | 0.40 | -0.54 | 0.0026 | 0.94 | 0.02 | 0.92 | -0.02 | - | 0.94 | 0.93 | -0.02 | NS |
| 36 | SIN3B_P607_F | Y | 19 | 0.83 | -0.08 | 0.27 | -0.62 | 0.0005 | 0.94 | 0.03 | 0.90 | 0.02 | - | 0.89 | 0.91 | 0.02 | NS |
| 37 | SOD3_P460_R | N | 4 | 0.30 | -0.44 | 0.19 | -0.57 | 0.0007 | 0.78 | 0.04 | 0.82 | 0.06 | - | 0.77 | 0.74 | -0.03 | NS |
| 38 | PLA2G2A_P528_F | N | 1 | 0.60 | -0.21 | 0.14 | -0.54 | 0.0001 | 0.89 | 0.08 | 0.46 | -0.21 | - | 0.68 | 0.81 | -0.13 | NS |
| 39 | PTHR1_P258_F | N | 3 | 0.66 | -0.08 | 0.14 | -0.49 | 0.0001 | 0.82 | 0.08 | 0.50 | -0.14 | - | 0.64 | 0.74 | 0.10 | NS |
| 40 | BCR_P346_F | Y | 22 | 0.39 | -0.44 | 0.64 | -0.28 | 0.0260 | 0.97 | 0.13 | 0.80 | -0.13 | - | 0.92 | 0.84 | -0.09 | NS |
| 41 | ERCC3_P1210_R | N | 2 | 0.53 | -0.26 | 0.21 | -0.69 | 0.0265 | 0.93 | 0.14 | 0.94 | 0.04 | - | 0.90 | 0.79 | -0.11 | NS |
| 42 | GNMT_E126_F | Y | 6 | 0.30 | 0.27 | 0.45 | 0.38 | NS | 0.23 | 0.20 | 0.51 | 0.45 | 0.0075 | 0.06 | 0.03 | -0.04 | 0.0214 |
| 43 | FRZB_E186_R | Y | 2 | 0.25 | 0.15 | 0.51 | 0.49 | NS | 0.67 | 0.58 | 0.69 | 0.67 | 0.0001 | 0.02 | 0.10 | 0.07 | 0.0109 |
| 44 | HTR1B_E232_R | Y | 6 | 0.34 | 0.24 | 0.68 | 0.65 | NS | 0.79 | 0.68 | 0.89 | 0.86 | 0.0000 | 0.03 | 0.10 | 0.07 | 0.0109 |
| 45 | MYOD1_E156_F | Y | 11 | 0.52 | 0.40 | 0.24 | 0.21 | NS | 0.54 | 0.43 | 0.72 | 0.69 | 0.0000 | 0.03 | 0.11 | 0.08 | 0.0109 |
| 46 | AXL_E61_F | N | 19 | 0.57 | 0.48 | 0.24 | 0.06 | NS | 0.46 | 0.38 | 0.63 | 0.45 | 0.0036 | 0.18 | 0.09 | -0.10 | NS |
| 47 | HS3ST2_E145_R | Y | 16 | 0.61 | 0.51 | 0.35 | 0.31 | NS | 0.92 | 0.82 | 0.89 | 0.86 | 0.0001 | 0.03 | 0.10 | 0.07 | 0.0164 |
| 48 | RAB32_P493_R | Y | 6 | 0.13 | 0.10 | 0.03 | -0.03 | - | 0.86 | 0.83 | 0.57 | 0.51 | 0.0001 | 0.06 | 0.03 | -0.03 | NS |
| 49 | NEFL_P209_R | Y | 8 | 0.25 | 0.15 | 0.26 | 0.21 | - | 0.89 | 0.78 | 0.83 | 0.78 | 0.0001 | 0.05 | 0.11 | 0.06 | 0.0109 |
| 50 | SFRP1_P157_F | Y | 8 | 0.04 | 0.01 | 0.08 | 0.05 | - | 0.77 | 0.74 | 0.65 | 0.62 | 0.0002 | 0.02 | 0.03 | 0.01 | NS |
| 51 | SLIT2_P208_F | Y | 4 | 0.37 | 0.32 | 0.32 | 0.29 | NS | 0.79 | 0.74 | 0.63 | 0.60 | 0.0001 | 0.03 | 0.05 | 0.02 | 0.0214 |
| 52 | PTPRG_E40_R | Y | 3 | 0.05 | 0.01 | 0.35 | 0.32 | - | 0.78 | 0.73 | 0.61 | 0.58 | 0.0001 | 0.03 | 0.04 | 0.01 | 0.0164 |
| 53 | DLK1_E227_R | Y | 14 | 0.26 | 0.20 | 0.28 | 0.25 | - | 0.78 | 0.73 | 0.74 | 0.71 | 0.0001 | 0.03 | 0.05 | 0.02 | 0.0331 |
| 54 | FAT_P279_R | Y | 4 | 0.17 | 0.07 | 0.36 | 0.32 | - | 0.83 | 0.73 | 0.72 | 0.67 | 0.0001 | 0.04 | 0.10 | 0.06 | 0.0109 |
| 55 | IGSF4_P86_R | Y | 11 | 0.14 | 0.08 | 0.08 | 0.05 | - | 0.77 | 0.72 | 0.45 | 0.42 | 0.0009 | 0.04 | 0.06 | 0.02 | NS |
| 56 | CHFR_P501_F | Y | 12 | 0.21 | 0.16 | 0.07 | 0.04 | - | 0.69 | 0.65 | 0.53 | 0.50 | 0.0000 | 0.03 | 0.05 | 0.02 | 0.0214 |
| 57 | IL17RB_E164_R | Y | 3 | 0.20 | 0.11 | 0.25 | 0.20 | - | 0.72 | 0.63 | 0.50 | 0.45 | 0.0013 | 0.05 | 0.09 | 0.04 | 0.0331 |
| 58 | SFRP1_E398_R | Y | 8 | 0.05 | 0.00 | 0.08 | 0.07 | - | 0.63 | 0.58 | 0.57 | 0.56 | 0.0005 | 0.02 | 0.05 | 0.04 | 0.0109 |
| 59 | HTR1B_P222_F | Y | 6 | 0.19 | 0.15 | 0.21 | 0.19 | - | 0.59 | 0.55 | 0.54 | 0.52 | 0.0001 | 0.02 | 0.04 | 0.02 | 0.0412 |

| 60 | ADAMTS12_E52_R | Y | 5 | 0.18 | 0.09 | 0.05 | 0.02 | - | 0.63 | 0.54 | 0.39 | 0.37 | 0.0002 | 0.02 | 0.09 | 0.07 | 0.0109 |
| --- | --- | --- | --- | --- | --- | --- | --- | --- | --- | --- | --- | --- | --- | --- | --- | --- | --- |
| 61 | SLC22A3_E122_R | Y | 6 | 0.11 | 0.02 | 0.19 | 0.14 | - | 0.62 | 0.53 | 0.60 | 0.54 | 0.0001 | 0.05 | 0.08 | 0.03 | NS |
| 62 | DCC_P471_R | Y | 18 | 0.24 | 0.16 | 0.26 | 0.21 | - | 0.60 | 0.52 | 0.54 | 0.49 | 0.0001 | 0.05 | 0.08 | 0.03 | 0.0300 |
| 63 | FGF12_P210_R | Y | 3 | 0.08 | 0.05 | 0.23 | 0.19 | - | 0.56 | 0.52 | 0.47 | 0.43 | 0.0033 | 0.05 | 0.03 | -0.01 | NS |
| 64 | CHGA_E52_F | Y | 14 | 0.10 | 0.03 | 0.10 | 0.06 | - | 0.59 | 0.52 | 0.50 | 0.46 | 0.0011 | 0.04 | 0.07 | 0.03 | 0.0435 |
| 65 | ITGA2_E120_F | Y | 5 | 0.02 | -0.01 | 0.11 | 0.09 | - | 0.55 | 0.52 | 0.59 | 0.57 | 0.0010 | 0.02 | 0.03 | 0.01 | NS |
| 66 | DKFZP564O0823_E45_F | Y | 4 | 0.05 | 0.01 | 0.07 | 0.04 | - | 0.51 | 0.48 | 0.54 | 0.51 | 0.0009 | 0.03 | 0.04 | 0.01 | NS |
| 67 | GABRB3_E42_F | Y | 15 | 0.20 | 0.15 | 0.12 | 0.09 | - | 0.47 | 0.42 | 0.47 | 0.44 | 0.0028 | 0.03 | 0.05 | 0.03 | 0.0109 |
| 68 | NGFB_P13_F | Y | 1 | 0.11 | 0.04 | 0.30 | 0.14 | - | 0.48 | 0.41 | 0.72 | 0.56 | 0.0003 | 0.16 | 0.08 | -0.08 | 0.0300 |
| 69 | WNT2_P217_F | Y | 7 | 0.29 | 0.21 | 0.25 | 0.20 | - | 0.48 | 0.40 | 0.63 | 0.58 | 0.0016 | 0.05 | 0.08 | 0.03 | 0.0164 |
| 70 | TAL1_P594_F | Y | 1 | 0.26 | 0.20 | 0.24 | 0.18 | - | 0.46 | 0.40 | 0.60 | 0.54 | 0.0001 | 0.06 | 0.06 | 0.00 | NS |
| 71 | PGF_P320_F | Y | 14 | 0.29 | 0.05 | 0.34 | 0.01 | - | 0.64 | 0.40 | 0.86 | 0.53 | 0.0003 | 0.33 | 0.24 | -0.09 | NS |
| 72 | CARD15_P302_R | N | 16 | 0.11 | -0.17 | 0.15 | -0.09 | - | 0.66 | 0.38 | 0.77 | 0.53 | 0.0001 | 0.24 | 0.28 | 0.04 | NS |
| 73 | ISL1_P379_F | Y | 5 | 0.26 | 0.20 | 0.21 | 0.20 | - | 0.41 | 0.35 | 0.45 | 0.43 | 0.0071 | 0.02 | 0.06 | 0.04 | 0.0109 |
| 74 | MME_E29_F | Y | 3 | 0.20 | 0.10 | 0.28 | 0.22 | - | 0.78 | 0.68 | 0.08 | 0.03 | 0.0017 | 0.05 | 0.10 | 0.05 | 0.0109 |
| 75 | DAPK1_P10_F | Y | 9 | 0.04 | -0.03 | 0.02 | 0.01 | - | 0.70 | 0.64 | 0.15 | 0.13 | 0.0002 | 0.02 | 0.06 | 0.04 | 0.0109 |
| 76 | IGFBP2_P306_F | Y | 2 | 0.02 | -0.01 | 0.02 | 0.00 | - | 0.65 | 0.63 | 0.25 | 0.23 | 0.0036 | 0.02 | 0.03 | 0.01 | NS |
| 77 | RAP1A_P285_R | Y | 1 | 0.20 | -0.09 | 0.45 | -0.04 | - | 0.87 | 0.57 | 0.81 | 0.32 | 0.0001 | 0.49 | 0.29 | -0.19 | 0.0372 |
| 78 | GADD45A_P737_R | N | 1 | 0.20 | 0.07 | 0.31 | -0.04 | - | 0.70 | 0.57 | 0.62 | 0.27 | 0.0013 | 0.34 | 0.13 | -0.21 | 0.0214 |
| 79 | NEFL_E23_R | Y | 8 | 0.38 | 0.23 | 0.53 | 0.12 | NS | 0.63 | 0.49 | 0.72 | 0.30 | 0.0161 | 0.41 | 0.14 | -0.27 | NS |
| 80 | CSF3R_P472_F | N | 1 | 0.31 | -0.16 | 0.38 | 0.07 | - | 0.78 | 0.31 | 0.88 | 0.57 | 0.0000 | 0.31 | 0.47 | 0.16 | NS |
| 81 | EGFR_E295_R | Y | 7 | 0.13 | 0.09 | 0.23 | 0.21 | - | 0.33 | 0.30 | 0.57 | 0.56 | 0.0034 | 0.02 | 0.03 | 0.01 | 0.0331 |
| 82 | KIAA1804_P689_R | Y | 1 | 0.08 | 0.05 | 0.18 | 0.12 | - | 0.31 | 0.28 | 0.52 | 0.47 | 0.0026 | 0.05 | 0.03 | -0.02 | NS |
| 83 | ISL1_E87_R | Y | 5 | 0.22 | 0.16 | 0.17 | 0.15 | - | 0.33 | 0.27 | 0.56 | 0.53 | 0.0065 | 0.03 | 0.06 | 0.03 | 0.0109 |
| 84 | NTRK3_P636_R | Y | 15 | 0.03 | 0.00 | 0.11 | 0.07 | - | 0.29 | 0.26 | 0.52 | 0.49 | 0.0059 | 0.03 | 0.03 | 0.00 | NS |
| 85 | BDNF_P259_R | Y | 11 | 0.13 | -0.09 | 0.27 | 0.15 | - | 0.45 | 0.23 | 0.70 | 0.57 | 0.0002 | 0.12 | 0.22 | 0.10 | 0.0372 |
| 86 | ZNF215_P71_R | Y | 11 | 0.24 | 0.08 | 0.33 | 0.16 | - | 0.37 | 0.21 | 0.71 | 0.54 | 0.0001 | 0.17 | 0.16 | -0.01 | NS |

| 91 | RARRES1_E235_F | Y | 3 | 0.14 | 0.12 | 0.07 | 0.06 | - | 0.19 | 0.16 | 0.44 | 0.43 | 0.0075 | 0.02 | 0.03 | 0.01 | 0.0164 |
| --- | --- | --- | --- | --- | --- | --- | --- | --- | --- | --- | --- | --- | --- | --- | --- | --- | --- |
| 92 | MEST_P62_R | Y | 7 | 0.26 | 0.20 | 0.30 | 0.21 | - | 0.21 | 0.15 | 0.58 | 0.49 | 0.0070 | 0.09 | 0.06 | -0.03 | NS |
| 93 | KCNK4_E3_F | Y | 11 | 0.36 | 0.12 | 0.57 | 0.26 | - | 0.13 | -0.11 | 0.76 | 0.46 | 0.0001 | 0.30 | 0.24 | -0.06 | NS |
| 94 | MET_E333_F | Y | 7 | 0.36 | -0.12 | 0.23 | -0.09 | - | 0.16 | -0.31 | 0.66 | 0.34 | 0.0138 | 0.33 | 0.48 | 0.15 | NS |
| 95 | LCK_E28_F | Y | 1 | 0.89 | 0.07 | 0.63 | -0.22 | - | 0.30 | -0.51 | 0.09 | -0.76 | 0.0000 | 0.85 | 0.81 | -0.04 | NS |
| 96 | SFTPB_P689_R | N | 2 | 0.84 | -0.03 | 0.55 | -0.21 | - | 0.14 | -0.73 | 0.13 | -0.63 | 0.0001 | 0.76 | 0.87 | 0.11 | 0.0435 |
| 97 | LTA_P214_R | N | 6 | 0.80 | 0.10 | 0.49 | -0.31 | - | 0.11 | -0.58 | 0.19 | -0.61 | 0.0002 | 0.80 | 0.69 | -0.10 | NS |
| 98 | IL10_P348_F | N | 1 | 0.33 | -0.33 | 0.06 | -0.59 | NS | 0.15 | -0.51 | 0.49 | -0.15 | 0.0177 | 0.65 | 0.66 | 0.01 | NS |
| 99 | RAN_P581_R | Y | 12 | 0.48 | -0.19 | 0.15 | -0.58 | NS | 0.24 | -0.42 | 0.58 | -0.15 | 0.0179 | 0.73 | 0.66 | -0.07 | NS |
| 100 | CHD2_P451_F | N | 15 | 0.94 | 0.01 | 0.57 | -0.22 | - | 0.50 | -0.44 | 0.62 | -0.16 | 0.0424 | 0.78 | 0.94 | 0.16 | 0.0109 |
| 101 | CCL3_E53_R | N | 17 | 0.10 | -0.53 | 0.02 | -0.67 | NS | 0.06 | -0.56 | 0.03 | -0.66 | 0.0000 | 0.69 | 0.62 | -0.07 | NS |
| 102 | BLK_P14_F | N | 8 | 0.62 | -0.18 | 0.18 | -0.57 | NS | 0.07 | -0.73 | 0.03 | -0.73 | 0.0000 | 0.76 | 0.80 | 0.04 | NS |
| 103 | MPL_P657_F | N | 1 | 0.47 | -0.13 | 0.35 | -0.36 | - | 0.06 | -0.54 | 0.21 | -0.50 | 0.0003 | 0.71 | 0.60 | -0.11 | NS |
| 104 | CTGF_P693_R | N | 6 | 0.86 | -0.11 | 0.59 | -0.35 | - | 0.33 | -0.64 | 0.51 | -0.43 | 0.0050 | 0.94 | 0.97 | 0.03 | 0.0164 |
| 105 | SLC22A18_P216_R | N | 11 | 0.26 | -0.21 | 0.05 | -0.69 | - | 0.12 | -0.35 | 0.20 | -0.55 | 0.0013 | 0.74 | 0.47 | -0.27 | NS |

*Probes numbered 1 to 41 correspond to the differentially methylated CpGs in AML MLL leukemia. Mean ß values of each selected CpG locus from each group of samples (primary and HSPC-MA9) and controls were estimated. FDR was correct for multiple testing in the ANOVA (^#^FDR) or the t test (^##^FDR). An FDR<0.05 was considered statistically significant.* ^*^*Indicates whether the selected CpG is included (Y) or not (N) in a CpG island.^**^Δß=(Mean primary samples ß value) – (Mean BM controls ß value).* *^***^Δß=(Mean HSPC-MA9 ß value) – (Mean CB controls ß value). ^****^Δß=(Mean BM controls ß value) – (Mean CB controls ß value). dUMCpGs are underlined.*
