# Supplementary material for: DNA Methylation Profiles and Their Relationship with Cytogenetic Status in Adult Acute Myeloid Leukemia
Source: PLoS One. 2010 Aug 16;5(8):e12197. doi: 10.1371/journal.pone.0012197 (PMC2922373; doi:10.1371/journal.pone.0012197)
Supplement: Table S6 — Methylation status of 81 CpGs selected as differentially methylated between primary CBF leukemia cases or HSPC-CBF samples and controls. (0.36 MB DOC) [file pone.0012197.s007.doc]

|  |  |  |  |  |  |  | Primary AE | | | HSPC-AE | | |  | Primary CM | | | | HSPC-CM | | |  | | | BM | | CB |  | |  | |
| --- | --- | --- | --- | --- | --- | --- | --- | --- | --- | --- | --- | --- | --- | --- | --- | --- | --- | --- | --- | --- | --- | --- | --- | --- | --- | --- | --- | --- | --- | --- |
|  |  |  |  |  |  |  |  | | |  | | |  |  | | | |  | | |  | | |  | |  | *^****^* Δß  | |  | |
|  | PROBE | | *^*^*CpG island | | CHR | | Mean β | *^**^* Δß | | Mean β | *^***^* Δß  | | *^#^*FDR | Mean β | | *^**^* Δß  | | Mean β | *^***^* Δß  | | *^#^*FDR | | | Mean β | | Mean β |  |  | *^##^*FDR | |
|  |  |  |  |  |  |  | values |  |  | values |  |  |  | values | |  |  | values |  |  |  |  |  | values | | values |  |  |  |  |
|  |  |  |  |  |  |  |  |  |  |  |  |  |  |  |  |  |  |  |  |  |  |  |  |  |  |  |  |  |  |  |
| 1 | EYA4_E277_F | | Y | | 6 | | 0.69 | 0.62 | 0.12 | | 0.03 | 0.0001 | | 0.31 | 0.24 | | 0.09 | | 0.00 | | 0.0043 | | 0.06 | | 0.09 | | 0.02 | | 0.0230 | |
| 2 | PITX2_E24_R | | Y | | 4 | | 0.74 | 0.60 | 0.22 | | 0.16 | 0.0001 | | 0.47 | 0.33 | | 0.11 | | 0.06 | | 0.0030 | | 0.14 | | 0.06 | | -0.08 | | 0.0080 | |
| 3 | SFRP1_P157_F | | Y | | 8 | | 0.62 | 0.59 | 0.04 | | 0.02 | 0.0000 | | 0.27 | 0.24 | | 0.02 | | 0.00 | | 0.0120 | | 0.03 | | 0.02 | | -0.01 | | NS | |
| 4 | CDKN2Bseq50S294_F | | Y | | 9 | | 0.62 | 0.57 | 0.02 | | -0.01 | 0.0001 | | 0.33 | 0.29 | | 0.02 | | -0.01 | | 0.0025 | | 0.05 | | 0.03 | | -0.01 | | NS | |
| 5 | SLC22A3_E122_R | | Y | | 6 | | 0.65 | 0.56 | 0.09 | | 0.04 | 0.0001 | | 0.23 | 0.15 | | 0.07 | | 0.01 | | NS | | 0.08 | | 0.05 | | -0.03 | | NS | |
| 6 | NGFB_P13_F | | Y | | 1 | | 0.64 | 0.56 | 0.17 | | 0.01 | 0.0001 | | 0.16 | 0.08 | | 0.13 | | -0.03 | | NS | | 0.08 | | 0.16 | | 0.08 | | 0.0270 | |
| 7 | SFRP1_E398_R | | Y | | 8 | | 0.60 | 0.54 | 0.02 | | 0.01 | 0.0000 | | 0.24 | 0.19 | | 0.02 | | 0.00 | | 0.0250 | | 0.05 | | 0.02 | | -0.04 | | 0.0080 | |
| 8 | NGFB_E353_F | | Y | | 1 | | 0.58 | 0.53 | 0.03 | | 0.00 | 0.0002 | | 0.15 | 0.10 | | 0.02 | | 0.00 | | 0.0120 | | 0.05 | | 0.02 | | -0.03 | | 0.0080 | |
| 9 | ETV1_P235_F | | N | | 7 | | 0.62 | 0.52 | 0.07 | | 0.02 | 0.0001 | | 0.31 | 0.22 | | 0.07 | | 0.01 | | 0.0034 | | 0.10 | | 0.06 | | -0.04 | | 0.0300 | |
| 10 | TUSC3_E29_R | | Y | | 8 | | 0.56 | 0.50 | 0.04 | | 0.00 | 0.0002 | | 0.32 | 0.27 | | 0.03 | | -0.01 | | 0.0003 | | 0.05 | | 0.04 | | -0.02 | | NS | |
| 11 | HS3ST2_P171_F | | Y | | 16 | | 0.54 | 0.48 | 0.06 | | 0.02 | 0.0007 | | 0.29 | 0.23 | | 0.04 | | 0.00 | | 0.0110 | | 0.06 | | 0.04 | | -0.02 | | 0.0230 | |
| 12 | ADAMTS12_E52_R | | Y | | 5 | | 0.56 | 0.47 | 0.03 | | 0.01 | 0.0002 | | 0.29 | 0.20 | | 0.03 | | 0.00 | | 0.0060 | | 0.09 | | 0.02 | | -0.07 | | 0.0080 | |
| 13 | IGSF4_P86_R | | Y | | 11 | | 0.53 | 0.47 | 0.04 | | 0.00 | 0.0009 | | 0.30 | 0.25 | | 0.05 | | 0.02 | | 0.0099 | | 0.06 | | 0.04 | | -0.02 | | NS | |
| 14 | WNT2_P217_F | | Y | | 7 | | 0.54 | 0.46 | 0.06 | | 0.01 | 0.0002 | | 0.35 | 0.28 | | 0.05 | | 0.00 | | 0.0110 | | 0.08 | | 0.05 | | -0.03 | | 0.0140 | |
| 15 | ISL1_P379_F | | Y | | 5 | | 0.52 | 0.46 | 0.02 | | 0.00 | 0.0003 | | 0.25 | 0.19 | | 0.02 | | 0.00 | | 0.0130 | | 0.06 | | 0.02 | | -0.04 | | 0.0080 | |
| 16 | DCC_P471_R | | Y | | 18 | | 0.53 | 0.45 | 0.07 | | 0.02 | 0.0003 | | 0.35 | 0.27 | | 0.07 | | 0.03 | | 0.0009 | | 0.08 | | 0.05 | | -0.03 | | 0.0270 | |
| 17 | PENK_P447_R | | Y | | 8 | | 0.51 | 0.44 | 0.11 | | 0.06 | 0.0003 | | 0.35 | 0.28 | | 0.07 | | 0.02 | | 0.0020 | | 0.07 | | 0.05 | | -0.02 | | NS | |
| 18 | IGFBP3_P423_R | | Y | | 7 | | 0.47 | 0.43 | 0.03 | | 0.01 | 0.0008 | | 0.27 | 0.23 | | 0.07 | | 0.05 | | 0.0170 | | 0.04 | | 0.02 | | -0.02 | | 0.0190 | |
| 19 | SOX17_P287_R | | Y | | 8 | | 0.58 | 0.42 | 0.12 | | 0.00 | 0.0002 | | 0.42 | 0.25 | | 0.18 | | 0.07 | | 0.0093 | | 0.17 | | 0.11 | | -0.06 | | NS | |
| 20 | IL17RB_E164_R | | Y | | 3 | | 0.51 | 0.42 | 0.09 | | 0.04 | 0.0006 | | 0.32 | 0.23 | | 0.03 | | -0.02 | | 0.0060 | | 0.09 | | 0.05 | | -0.04 | | 0.0300 | |
| 21 | PENK_E26_F | | Y | | 8 | | 0.52 | 0.41 | 0.07 | | 0.01 | 0.0001 | | 0.43 | 0.32 | | 0.06 | | 0.00 | | 0.0015 | | 0.11 | | 0.06 | | -0.04 | | 0.0080 | |
| 22 | GALR1_E52_F | | Y | | 18 | | 0.45 | 0.41 | 0.05 | | 0.02 | 0.0012 | | 0.35 | 0.31 | | 0.03 | | 0.00 | | 0.0034 | | 0.04 | | 0.03 | | -0.01 | | 0.0300 | |
| 23 | SLC5A8_E60_R | | Y | | 12 | | 0.56 | 0.39 | 0.08 | | 0.01 | 0.0001 | | 0.47 | 0.30 | | 0.07 | | 0.01 | | 0.0009 | | 0.17 | | 0.07 | | -0.10 | | 0.0080 | |
| 24 | DCC_E53_R | | Y | | 18 | | 0.64 | 0.39 | 0.48 | | 0.34 | 0.0008 | | 0.48 | 0.23 | | 0.11 | | -0.04 | | 0.0015 | | 0.25 | | 0.15 | | -0.10 | | 0.0230 | |
| 25 | FAT_P279_R | | Y | | 4 | | 0.47 | 0.37 | 0.04 | | 0.00 | 0.0018 | | 0.31 | 0.21 | | 0.04 | | 0.00 | | 0.0110 | | 0.10 | | 0.04 | | -0.06 | | 0.0080 | |
| 26 | NEFL_P209_R | | Y | | 8 | | 0.72 | 0.61 | 0.15 | | 0.10 | 0.0000 | | 0.48 | 0.37 | | 0.06 | | 0.01 | | 0.0003 | | 0.11 | | 0.05 | | -0.06 | | 0.0080 | |
| 27 | MME_E29_F | | Y | | 3 | | 0.64 | 0.54 | 0.05 | | 0.00 | 0.0001 | | 0.47 | 0.37 | | 0.04 | | -0.01 | | 0.0006 | | 0.10 | | 0.05 | | -0.05 | | 0.0080 | |
| 28 | ZNF215_P71_R | | Y | | 11 | | 0.61 | 0.45 | 0.19 | | 0.02 | 0.0002 | | 0.60 | 0.44 | | 0.18 | | 0.01 | | 0.0001 | | 0.16 | | 0.17 | | 0.01 | | NS | |
| 29 | HTR1B_P222_F | | Y | | 6 | | 0.45 | 0.40 | 0.09 | | 0.07 | 0.0010 | | 0.43 | 0.39 | | 0.04 | | 0.02 | | 0.0004 | | 0.04 | | 0.02 | | -0.02 | | 0.0370 | |
| 30 | KCNK4_E3_F | | Y | | 11 | | 0.62 | 0.37 | 0.83 | | 0.53 | 0.0001 | | 0.45 | 0.20 | | 0.67 | | 0.36 | | 0.0074 | | 0.24 | | 0.30 | | 0.06 | | NS | |
| 31 | COL1A2_P407_R | | N | | 7 | | 0.70 | 0.19 | 0.64 | | 0.41 | 0.0025 | | 0.58 | 0.07 | | 0.23 | | 0.00 | | 0.0330 | | 0.51 | | 0.24 | | -0.27 | | 0.0230 | |
| 32 | CREB1_P819_F | | Y | | 2 | | 0.05 | -0.01 | 0.74 | | 0.36 | 0.0000 | | 0.07 | 0.01 | | 0.46 | | 0.08 | | 0.0015 | | 0.06 | | 0.38 | | 0.32 | | 0.0140 | |
| 33 | SEMA3B_P110_R | | N | | 3 | | 0.04 | -0.07 | 0.76 | | 0.41 | 0.0000 | | 0.06 | -0.05 | | 0.63 | | 0.27 | | 0.0000 | | 0.11 | | 0.36 | | 0.25 | | 0.0140 | |
| 34 | MST1R_P392_F | | Y | | 3 | | 0.03 | 0.00 | 0.68 | | 0.45 | 0.0000 | | 0.08 | 0.05 | | 0.58 | | | 0.35 | | 0.0003 | 0.03 | | 0.23 | | 0.20 | | 0.0080 | |
| 35 | MFAP4_P10_R | | N | | 17 | | 0.07 | -0.03 | 0.63 | | 0.42 | 0.0000 | | 0.09 | -0.01 | | 0.70 | | | 0.49 | | 0.0000 | 0.11 | | 0.21 | | 0.10 | | NS | |
| 36 | RARA_P176_R | | N | | 17 | | 0.15 | -0.13 | 0.75 | | 0.54 | 0.0000 | | 0.52 | 0.24 | | 0.68 | | | 0.47 | | 0.0022 | 0.28 | | 0.21 | | -0.07 | | NS | |
| 37 | CD9_P585_R | | Y | | 12 | | 0.23 | -0.13 | 0.78 | | 0.50 | 0.0000 | | 0.20 | -0.15 | | 0.74 | | | 0.46 | | 0.0000 | 0.36 | | 0.28 | | -0.07 | | NS | |
| 38 | IL10_P85_F | | N | | 1 | | 0.07 | -0.13 | 0.60 | | 0.51 | 0.0000 | | 0.07 | -0.14 | | 0.70 | | | 0.61 | | 0.0000 | 0.21 | | 0.10 | | -0.11 | | NS | |
| 39 | MST1R_E42_R | | Y | | 3 | | 0.43 | -0.37 | 0.93 | | 0.05 | 0.0000 | | 0.58 | -0.22 | | 0.92 | | | 0.05 | | 0.0051 | 0.80 | | 0.87 | | 0.07 | | NS | |
| 40 | MAP3K8_P1036_F | | Y | | 10 | | 0.38 | -0.42 | 0.95 | | 0.03 | 0.0001 | | 0.66 | -0.15 | | 0.95 | | | 0.03 | | 0.0074 | 0.80 | | 0.92 | | 0.12 | | NS | |
| 41 | MPO_E302_R | | N | | 17 | | 0.30 | -0.43 | 0.95 | | 0.30 | 0.0000 | | 0.30 | -0.43 | | 0.91 | | | 0.26 | | 0.0000 | 0.73 | | 0.65 | | -0.08 | | NS | |
| 42 | SLC22A18_P472_R | | N | | 11 | | 0.34 | -0.48 | 0.85 | | -0.05 | 0.0000 | | 0.45 | -0.36 | | 0.91 | | | 0.01 | | 0.0000 | 0.81 | | 0.90 | | 0.09 | | NS | |
| 43 | HOXB2_P488_R | | N | | 17 | | 0.90 | 0.14 | 0.43 | | -0.34 | 0.0000 | | 0.61 | -0.16 | | 0.10 | | | -0.67 | | 0.0000 | 0.77 | | 0.77 | | 0.00 | | NS | |
| 44 | CDH13_E102_F | | Y | | 16 | | 0.66 | 0.61 | 0.07 | | 0.03 | 0.0000 | | 0.29 | 0.24 | | 0.04 | | | 0.00 | | 0.0050 | 0.05 | | 0.04 | | -0.02 | | 0.0440 | |
| 45 | DIO3_P674_F | | Y | | 14 | | 0.60 | 0.57 | 0.04 | | 0.02 | 0.0000 | | 0.30 | 0.27 | | 0.06 | | | 0.04 | | 0.0045 | 0.03 | | 0.02 | | -0.01 | | 0.0080 | |
| 46 | CDH13_P88_F | | Y | | 16 | | 0.73 | 0.56 | 0.17 | | 0.05 | 0.0000 | | 0.48 | 0.32 | | 0.09 | | | -0.03 | | 0.0002 | 0.17 | | 0.12 | | -0.05 | | NS | |
| 47 | SOX1_P294_F | | Y | | 13 | | 0.61 | 0.53 | 0.03 | | 0.00 | 0.0002 | | 0.34 | 0.26 | | 0.04 | | | 0.00 | | 0.0022 | 0.08 | | 0.04 | | -0.04 | | 0.0080 | |
| 48 | SLIT2_P208_F | | Y | | 4 | | 0.55 | 0.50 | 0.04 | | 0.02 | 0.0004 | | 0.24 | 0.19 | | 0.05 | | | 0.02 | | 0.0073 | 0.05 | | 0.03 | | -0.02 | | 0.0190 | |
| 49 | CDH11_P354_R | | Y | | 16 | | 0.79 | 0.50 | 0.10 | | 0.04 | 0.0000 | | 0.62 | 0.33 | | 0.06 | | | 0.00 | | 0.0001 | 0.29 | | 0.07 | | -0.22 | | 0.0080 | |
| 50 | NEFL_E23_R | | Y | | 8 | | 0.63 | 0.49 | 0.26 | | -0.15 | 0.0110 | | 0.33 | 0.19 | | 0.42 | | | 0.01 | | NS | 0.14 | | 0.41 | | 0.27 | | NS | |
| 51 | EYA4_P794_F | | Y | | 6 | | 0.51 | 0.46 | 0.06 | | 0.04 | 0.0002 | | 0.24 | 0.20 | | 0.03 | | | 0.01 | | 0.0130 | 0.05 | | 0.02 | | -0.03 | | 0.0080 | |
| **52** | **DBC1_P351_R** | | Y | | 9 | | 0.81 | 0.76 | 0.02 | | 0.00 | 0.0000 | | 0.72 | 0.68 | | 0.02 | | | 0.00 | | 0.0000 | 0.05 | | 0.02 | | -0.03 | | 0.0140 | |
| 53 | HS3ST2_E145_R | | Y | | 16 | | 0.83 | 0.73 | 0.08 | | 0.05 | 0.0000 | | 0.71 | 0.61 | | 0.08 | | | 0.04 | | 0.0000 | 0.10 | | 0.03 | | -0.07 | | 0.0140 | |
| 54 | MOS_E60_R | | Y | | 8 | | 0.73 | 0.65 | 0.27 | | 0.21 | 0.0001 | | 0.60 | 0.52 | | 0.06 | | | -0.01 | | 0.0001 | 0.08 | | 0.07 | | -0.01 | | NS | |
| 55 | MYOD1_E156_F | | Y | | 11 | | 0.74 | 0.63 | 0.29 | | 0.26 | 0.0000 | | 0.50 | 0.39 | | 0.02 | | | -0.01 | | 0.0009 | 0.11 | | 0.03 | | -0.08 | | 0.0080 | |
| **56** | **DBC1_E204_F** | | Y | | 9 | | 0.74 | 0.58 | 0.16 | | 0.11 | 0.0000 | | 0.67 | 0.50 | | 0.10 | | | 0.05 | | 0.0000 | 0.17 | | 0.05 | | -0.11 | | 0.0080 | |
| 57 | HTR1B_E232_R | | Y | | 6 | | 0.65 | 0.54 | 0.06 | | 0.03 | 0.0002 | | 0.61 | 0.51 | | 0.15 | | | 0.12 | | 0.0005 | 0.10 | | 0.03 | | -0.07 | | 0.0080 | |
| 58 | ALOX12_P223_R | | Y | | 17 | | 0.72 | 0.46 | 0.94 | | 0.22 | 0.0064 | | 0.81 | 0.54 | | 0.94 | | | 0.22 | | 0.0034 | 0.27 | | 0.72 | | 0.46 | | NS | |
| 59 | FRZB_E186_R | | Y | | 2 | | 0.53 | 0.43 | 0.04 | | 0.02 | 0.0006 | | 0.70 | 0.60 | | 0.02 | | | 0.00 | | 0.0000 | 0.10 | | 0.02 | | -0.07 | | 0.0080 | |
| 60 | FZD9_E458_F | | Y | | 7 | | 0.52 | 0.42 | 0.15 | | 0.10 | 0.0014 | | 0.70 | 0.60 | | 0.05 | | | 0.00 | | 0.0000 | 0.10 | | 0.05 | | -0.05 | | 0.0080 | |
| 61 | ALOX12_E85_R | | Y | | 17 | | 0.74 | 0.39 | 0.96 | | 0.26 | 0.0062 | | 0.81 | 0.46 | | 0.96 | | | 0.26 | | 0.0048 | 0.35 | | 0.70 | | 0.35 | | NS | |
| 62 | HIC1_seq_48_S103_R | | Y | | 17 | | 0.83 | 0.59 | 0.92 | | 0.51 | 0.0007 | | 0.76 | 0.52 | | 0.61 | | | 0.20 | | 0.0120 | 0.24 | | 0.40 | | 0.16 | | NS | |
| 63 | MEST_P4_F | | Y | | 7 | | 0.23 | 0.19 | 0.63 | | 0.54 | 0.0023 | | 0.15 | 0.10 | | 0.29 | | | 0.20 | | 0.0460 | 0.05 | | 0.10 | | 0.05 | | NS | |
| 64 | GNMT_P197_F | | Y | | 6 | | 0.27 | 0.13 | 0.91 | | 0.57 | 0.0001 | | 0.31 | 0.17 | | 0.65 | | | 0.31 | | 0.0022 | 0.14 | | 0.35 | | 0.21 | | NS | |
| 65 | GNMT_E126_F | | Y | | 6 | | 0.07 | 0.05 | 0.84 | | 0.77 | 0.0001 | | 0.04 | 0.02 | | 0.21 | | | 0.14 | | 0.0160 | 0.03 | | 0.06 | | 0.04 | | 0.0190 | |
| 66 | AXL_E61_F | | N | | 19 | | 0.04 | -0.05 | 0.69 | | 0.50 | 0.0000 | | 0.39 | 0.30 | | 0.36 | | | 0.18 | | NS | 0.09 | | 0.18 | | | 0.10 | | NS |
| 67 | IGFBP1_P12_R | | Y | | 7 | | 0.54 | 0.17 | 0.50 | | 0.28 | NS | | 0.66 | 0.29 | | 0.57 | | | 0.36 | | 0.0110 | 0.37 | | 0.22 | | | -0.16 | | 0.0080 |
| 68 | AOC3_P890_R | | N | | 17 | | 0.17 | -0.42 | 0.76 | | 0.01 | 0.0000 | | 0.37 | -0.22 | | 0.82 | | | 0.07 | | 0.0000 | 0.59 | | 0.75 | | | 0.17 | | NS |
| 69 | TM7SF3_P1068_R | | N | | 12 | | 0.20 | -0.43 | 0.90 | | 0.12 | 0.0000 | | 0.43 | -0.19 | | 0.92 | | | 0.13 | | 0.0005 | 0.62 | | 0.78 | | | 0.16 | | 0.1800 |
| 70 | IL10_P348_F | | N | | 1 | | 0.23 | -0.43 | 0.94 | | 0.29 | 0.0000 | | 0.32 | -0.34 | | 0.95 | | | 0.30 | | 0.0000 | 0.66 | | 0.65 | | | -0.01 | | NS |
| 71 | PTK6_E50_F | | Y | | 20 | | 0.11 | -0.45 | 0.86 | | 0.06 | 0.0000 | | 0.32 | -0.25 | | 0.88 | | | 0.07 | | 0.0001 | 0.57 | | 0.80 | | | 0.24 | | 0.0340 |
| 72 | NOTCH4_P938_F | | N | | 6 | | 0.30 | -0.49 | 0.87 | | 0.01 | 0.0000 | | 0.57 | -0.22 | | 0.89 | | | 0.03 | | 0.0018 | 0.78 | | 0.86 | | | 0.08 | | NS |
| 73 | SOD3_P225_F | | N | | 4 | | 0.38 | -0.50 | 0.94 | | 0.02 | 0.0000 | | 0.58 | -0.29 | | 0.95 | | | 0.02 | | 0.0001 | 0.87 | | 0.93 | | | 0.06 | | NS |
| 74 | DDR1_P332_R | | N | | 6 | | 0.23 | -0.60 | 0.77 | | -0.04 | 0.0000 | | 0.63 | -0.19 | | 0.82 | | | 0.01 | | 0.0330 | 0.83 | | 0.81 | | | -0.02 | | NS |
| 75 | HGF_E102_R | | N | | 7 | | 0.09 | -0.34 | 0.90 | | 0.17 | 0.0000 | | 0.08 | -0.36 | | 0.82 | | | 0.09 | | 0.0000 | 0.43 | | 0.73 | | | 0.30 | | 0.0470 |
| 76 | HPN_P823_F | | N | | 19 | | 0.13 | -0.36 | 0.92 | | 0.44 | 0.0000 | | 0.82 | 0.32 | | 0.77 | | | 0.30 | | 0.0006 | 0.49 | | 0.47 | | | -0.02 | | NS |
| 77 | SEPT9_P58_R | | Y | | 17 | | 0.47 | -0.39 | 0.74 | | -0.19 | 0.0006 | | 0.51 | -0.35 | | 0.84 | | | -0.10 | | 0.0015 | 0.86 | | 0.94 | | | 0.08 | | 0.0370 |
| 78 | CCL3_E53_R | | N | | 17 | | 0.22 | -0.40 | 0.85 | | 0.16 | 0.0000 | | 0.19 | -0.44 | | 0.49 | | | -0.20 | | 0.0001 | 0.62 | | 0.69 | | | 0.07 | | NS |
| 79 | SOD3_P460_R | | N | | 4 | | 0.25 | -0.48 | 0.85 | | 0.09 | 0.0000 | | 0.31 | -0.43 | | 0.74 | | | -0.03 | | 0.0000 | 0.74 | | 0.77 | | | 0.03 | | NS |
| 80 | CSF3_P309_R | | N | | 17 | | 0.16 | -0.50 | 0.80 | | 0.07 | 0.0000 | | 0.31 | -0.36 | | 0.71 | | | -0.03 | | 0.0001 | 0.66 | | 0.74 | | | 0.07 | | NS |
| 81 | KRT13_P676_F | | N | | 17 | | 0.20 | -0.55 | 0.63 | | -0.11 | 0.0001 | | 0.29 | -0.45 | | 0.90 | | | 0.15 | | 0.0000 | 0.74 | | 0.75 | | | 0.00 | | NS |

*Mean ß values of each selected CpG locus from each group of samples (primary and HSPC) and controls were estimated. FDR was applied to correct for multiple testing in the ANOVA (^#^FDR) or the t test (^##^FDR). An FDR<0.05 was considered statistically significant. *Indicates whether the selected CpG is included (Y) or not (N) in a CpG-island ^**^Δß=(Mean primary samples ß value) – (Mean BM controls ß value). ^***^Δß=(Mean HSPC-MA9 ß value) – (Mean CB controls ß value).*

*^****^ Δß=(Mean BM controls ß value) – (Mean CB controls ß value). dUMCpGs are underlined.*
