# Supplementary figures and images for: DNA Methylation Profiles and Their Relationship with Cytogenetic Status in Adult Acute Myeloid Leukemia
Source: PLoS One. 2010 Aug 16;5(8):e12197. doi: 10.1371/journal.pone.0012197 (PMC2922373; doi:10.1371/journal.pone.0012197)

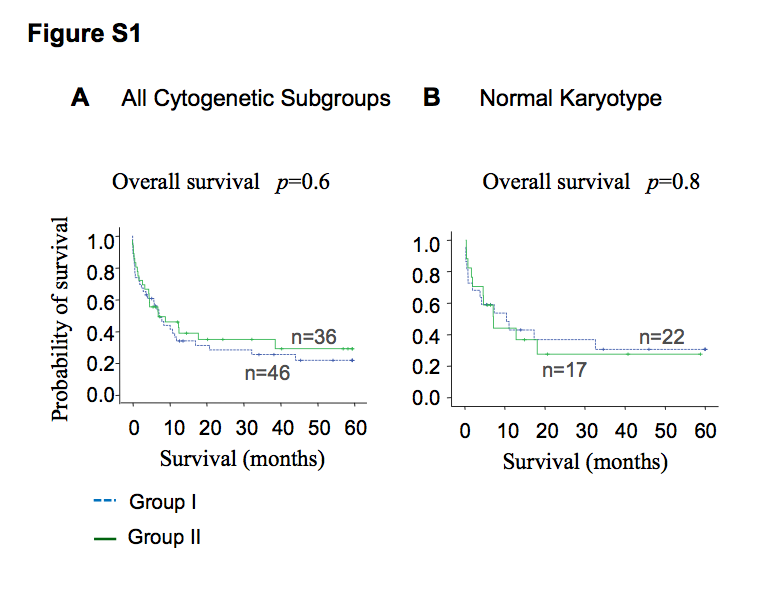

Supplement: Figure S1 — Kaplan-Meier curves for overall survival in cases with available clinical data stratified by methylation signature: A) All cases; B) Patients with a normal karyotype. (1.84 MB TIF) [file pone.0012197.s008.tif]

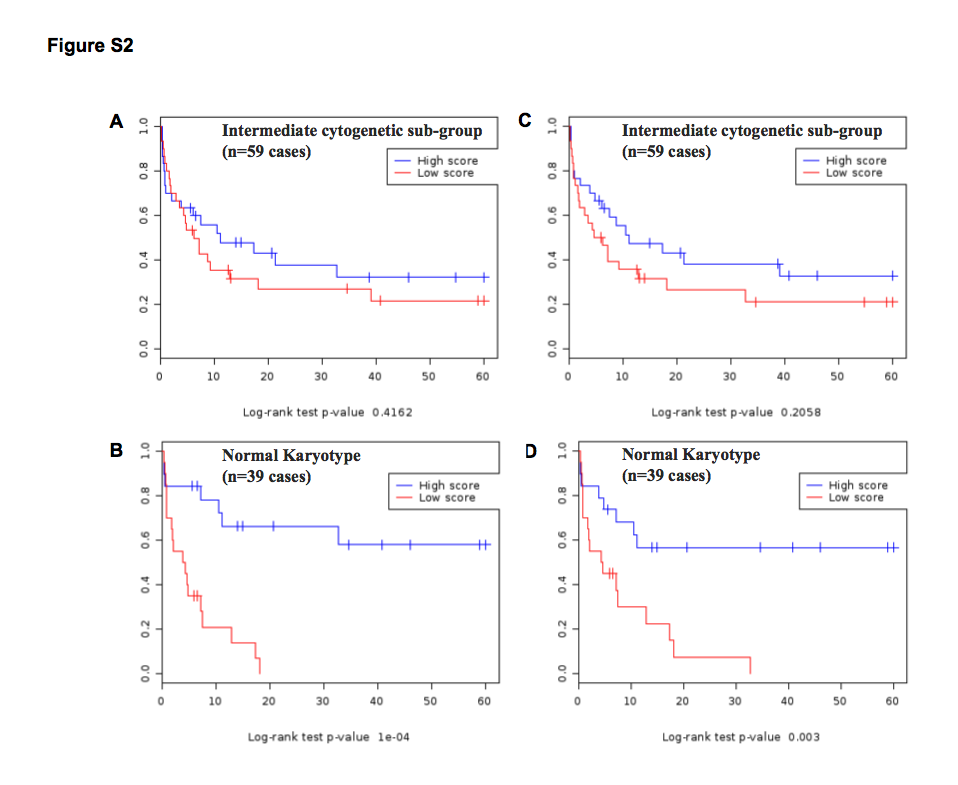

Supplement: Figure S2 — A DNA Methylation Classifier to predict Clinical Outcome in the AML cases included on the intermediate cytogenetic subgroup or in the Normal karyotype cases. Results obtained using the Beta values of the 115 CpGs with a larger variation across the original series, the SignS Web tool for gene selection and signature finding, build a predictor model of overall survival based on the methylation status of two CpGs, DBC1_E204_F and CDKN2B_sec50. Survival curves comparing low-score and high-score models from final models, with those two probes, using boosting of a component-wise Cox model (A and B) and the threshold gradient descent method (B and C) are shown. (3.01 MB TIF) [file pone.0012197.s009.tif]

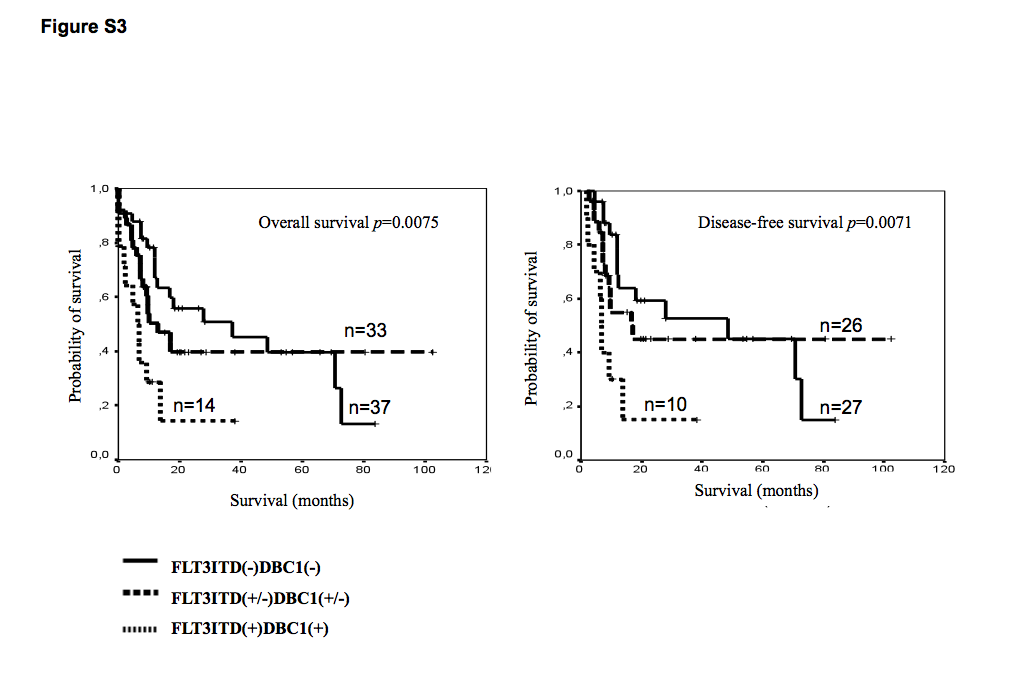

Supplement: Figure S3 — Kaplan-Meier overall survival and disease-free survival curves for patients with available clinical data and a normal karyotype at diagnosis from the validation series, stratified by MSP result and the DBC1 gene and the FLT3 status. (2.76 MB TIF) [file pone.0012197.s010.tif]

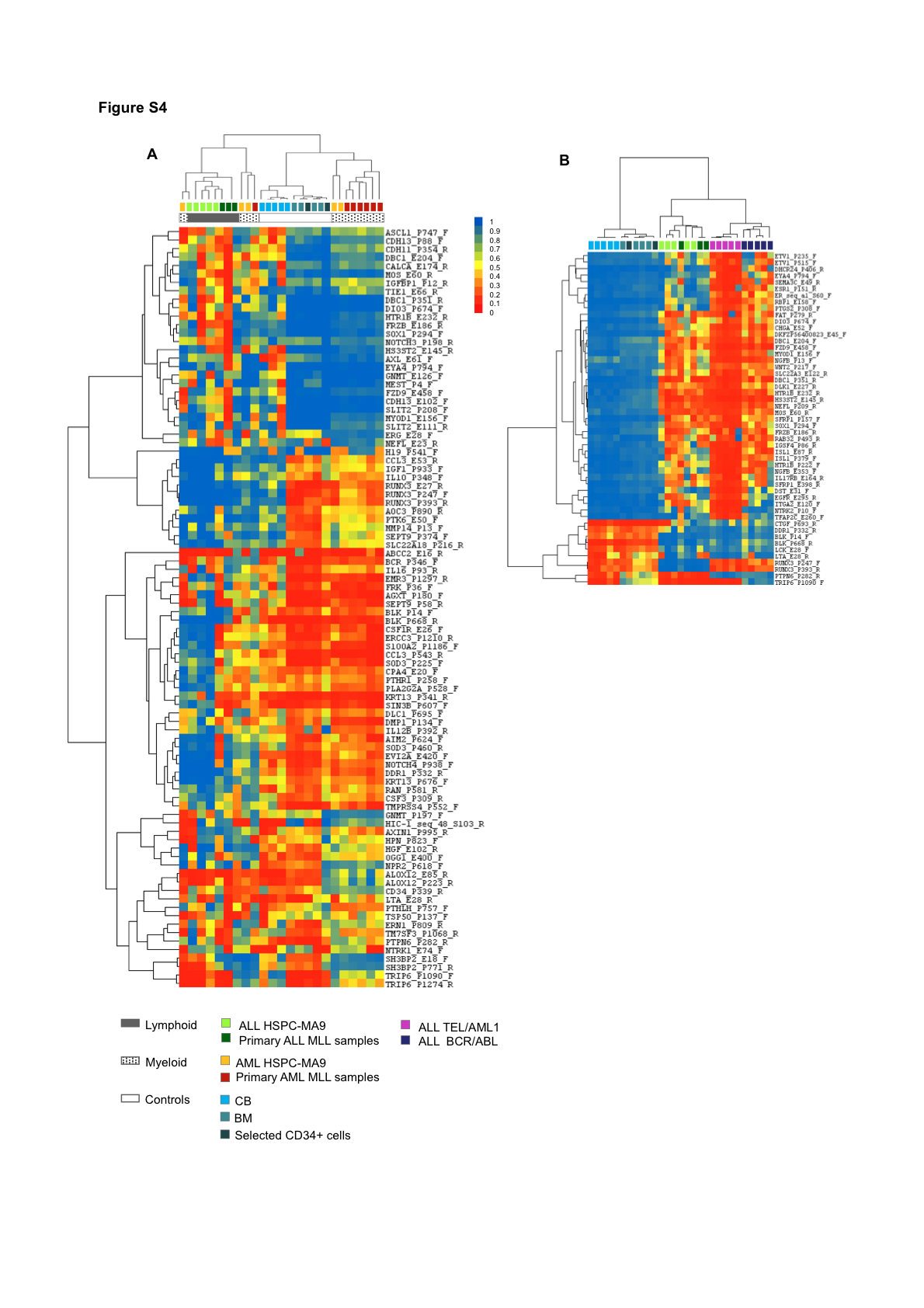

Supplement: Figure S4 — A) Unsupervised hierarchical clustering by applying the complete linkage method and uncentered based distance metric for 7 AML-primary MLL cases, 3 primary ALL-MLL cases, 10 HSPC-MA9 (5 myeloid and 5 lymphoid), and 11 controls (4 bone marrow, 2 selected CD34+, and 5 CB) in the 144 probes selected by filtering with a standard deviation over 0.25 across all samples. B) Unsupervised hierarchical clustering by applying the complete linkage method and uncentered based distance metric for 13 ALL primary cases (3 MLL cases, 5 TEL/AML1 cases, and 5 BCR/ABL cases), 5 lymphoid HSPC-MA9, and 11 controls (4 bone marrow, 2 selected CD34+ samples, and 5 cultured cord blood samples), using 51 probes with a standard deviation over 0.25 across all the samples. (6.01 MB TIF) [file pone.0012197.s011.tif]

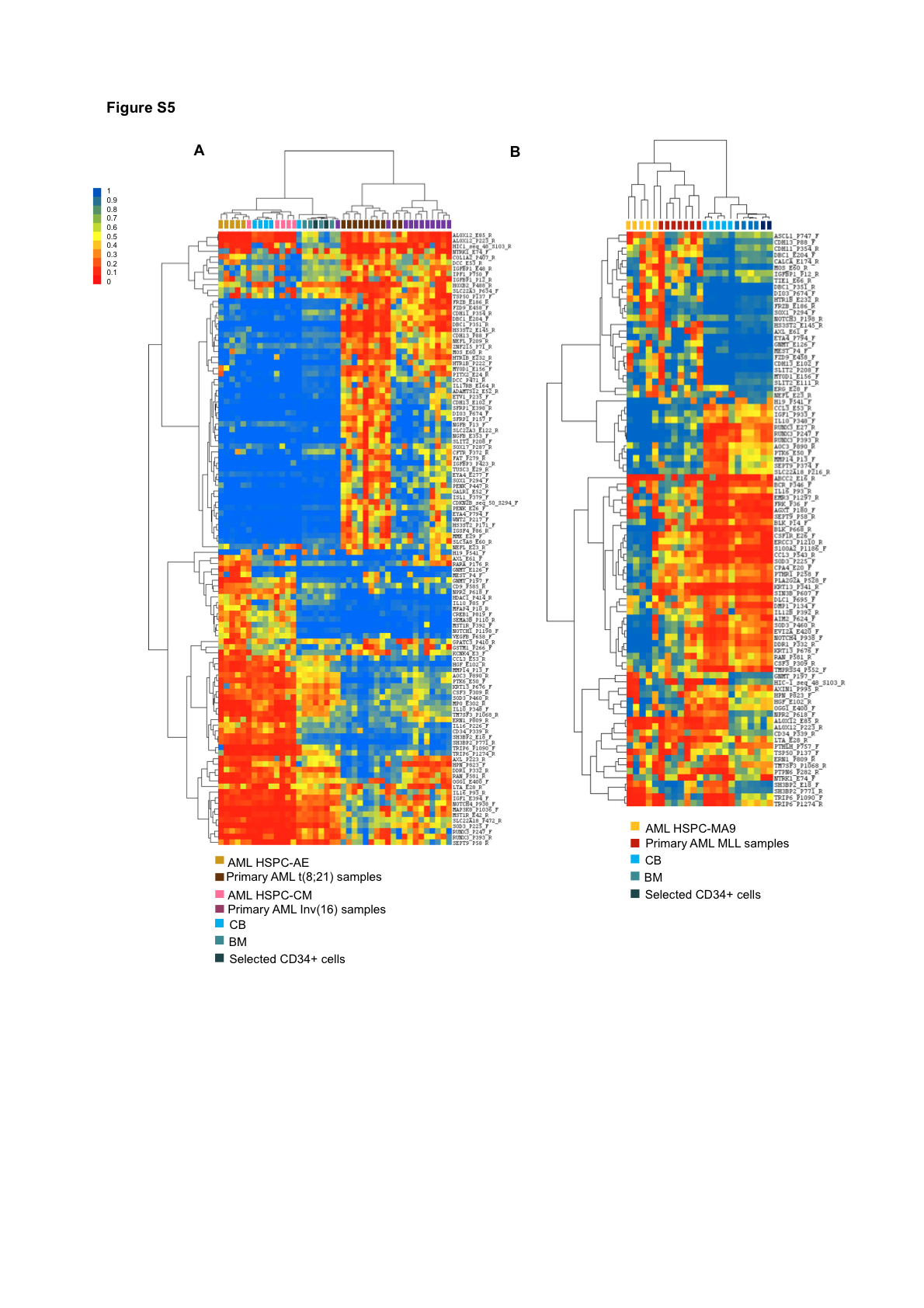

Supplement: Figure S5 — A) Unsupervised hierarchical clustering with the complete linkage method and euclidean-based distance metric of the primary CBF leukemia cases, HSPC-CBF and controls (bone marrow and cord blood). B) Unsupervised hierarchical clustering of the primary AML MLL leukemia cases, myeloid HSPC-MA9 and controls (bone marrow and cord blood) performed with 90 selected probes after filtering with an SD>0.25. (6.01 MB TIF) [file pone.0012197.s012.tif]
